# Antimicrobial Secondary Metabolites from the Seawater-Derived Fungus *Aspergillus sydowii* SW9

**DOI:** 10.3390/molecules24244596

**Published:** 2019-12-16

**Authors:** Yu-Jing Liu, Jian-Long Zhang, Chen Li, Xue-Gen Mu, Xiao-Li Liu, Lei Wang, Yan-Cui Zhao, Peng Zhang, Xiao-Dong Li, Xing-Xiao Zhang

**Affiliations:** 1 Key Laboratory of marine biotechnology in Universities of Shandong (Ludong University), School of Life Sciences, Ludong University, Yantai 264025, China; chunxiao6@163.com (Y.-J.L.); Zhangjianlong@ldu.edu.cn (J.-L.Z.); Lychees6601@163.com (C.L.); mxgdmn@163.com (X.-G.M.); lxlshz2006@163.com (X.-L.L.); wanglei9909@163.com (L.W.); yancuizh@aliyun.com (Y.-C.Z.); 2 Yantai Institute of Coastal Zone Research, Chinese Academy of Sciences, Yantai 264003, China; 3 Shandong Provincial Key Laboratory of Quality Safty Monitoring and Risk Assessment for Animal Products, Ji’nan 250022, China; 4 Tobacco Research Institute, Chinese Academy of Agricultural Sciences, Qingdao 266101, China; zhangpeng@caas.cn

**Keywords:** *Aspergillus sydowii*, seawater-derived fungus, secondary metabolites, antimicrobial activity

## Abstract

Marine-derived fungi are considered to be valuable producers of bioactive secondary metabolites used as lead compounds with medicinal importance. In this study, chemical investigation of the seawater-derived fungus *Aspergillus sydowii* SW9 led to the isolation and identification of one new quinazolinone alkaloid, 2-(4-hydroxybenzyl)-4-(3-acetyl)quinazolin-one (**1**), one new aromatic bisabolene-type sesquiterpenoid, (**2**) and one new chorismic acid analogue (**3**), as well as two known alkaloids (compounds **4** and **5**). Their structures were determined by extensive 1D/2D NMR and mass spectrometric data, and the absolute configurations of **2** and **3** were assigned by the analysis of ECD spectra aided by quantum chemical computations. Compounds **1**, **2,** and **4** exhibited selective inhibitory activities against the human pathogenic bacteria *Escherichia coli*, *Staphylococcus aureus*, *S. epidermidis,* and *Streptococcus pneumoniae*, with MIC values ranging from 2.0 to 16 μg/mL.

## 1. Introduction

The species *Aspergillus sydowii* is generally characterized as a causative agent of aspergillosis of sea fan corals, based on morphological, physiological and nucleotide sequence analysis [1,2,3]. On the other hand, *A. sydowii* is a biosynthetically talented fungal species with great potential to produce a wide range of structurally diversified secondary metabolites, such as alkaloids [4], polyketides [5,6,7,8,9], and sesquiterpenes [10,11,12,13], and more than half of these metabolites were obtained from marine-derived strains. Some metabolites isolated from *A. sydowii* exhibit attractive biological activities including antimicrobial [9], antioxidant [12], cytotoxic [6], and immunosuppressive [7], as well as protein tyrosine phosphatase-inhibiting activities [5,8]. Antibiotic active constituents from these marine-associated *A. sydowii* strains have been reported and caught our attention. For example, the new xanthones 2-hydroxy-1-(hydroxymethyl)-8-methoxy-3-methyl-9*H*-xanthen-9-one and 2-hydroxy-1-(hydroxymethyl)-7,8-dimethoxy-3-methyl-9*H*-xanthen-9-one displayed obvious inhibitory activity against influenza A virus H1N1, with IC_50_ values ranging from 2.17 to 4.70 μM [14], while β-d-glucopyranosyl aspergillusene A showed antibacterial activity against the human pathogen *Staphylococcus aureus* and fish pathogens *Streptococcus iniae* and *Vibrio ichthyoenteri* [15].

**Figure 1:**
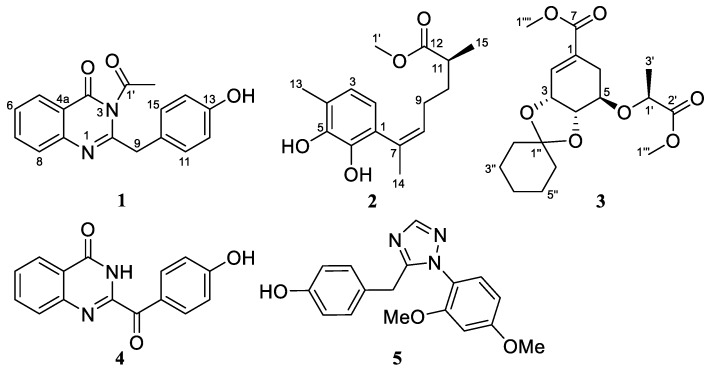
The structures of compounds **1**–**5** isolated from *Aspergillus sydowii SW9*.

With the purpose of further searching for new antibiotic active metabolites from *A. sydowii*, we continued our studies, and five compounds, including three previously undescribed compounds (**1**–**3**) along with two known alkaloids (**4**, **5**) (Figure 1), were obtained and identified from the culture extract of *A. sydowii* SW9. Their structures were identified by extensive 1D/2D NMR and mass spectrometric data, and the absolute configurations of the new compounds **2** and **3** were assigned by the analysis of ECD spectra aided by quantum chemical computations. All of these compounds were tested for antimicrobial activities against four human pathogenic bacterial strains.

## 2. Results and Discussion

**Figure 2:**
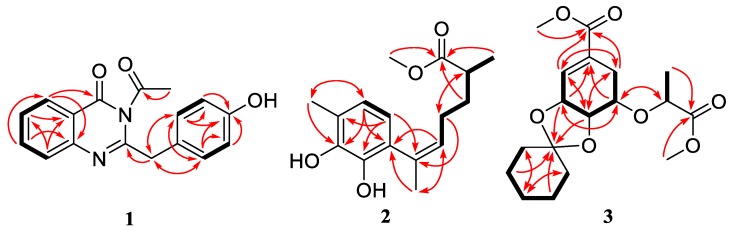
Key COSY (bold lines) and HMBC (red arrows) correlations for compounds **1**–**3**.

Compound **1** was obtained as white powder. Its molecular formula was determined as C_17_H_14_N_2_O_3_ by the HRESIMS at *m/z* 295.1079 [M + H]^+^ (Figure S1 in the Supplementary File), implying 12 degrees of unsaturation. The ^1^H-NMR spectrum (Table 1 and Figure S2) displayed signals for eight aromatic protons in the downfield region. The ^13^C-NMR and DEPT data (Table 1 and Figure S3) exhibited 17 carbon signals classified as one methyl, one methylene, eight methines, and seven quaternary carbons. Analysis of the ^1^H-^1^H coupling patterns (Figure 2 and Figure S4) revealed an *o*-substituted aryl ring, and the remaining four aromatic protons were attributed to a *p*-substituted aryl ring with two sets of doublets (*J* = 7.8 Hz each) at *δ*_H_ 6.70 and 7.16 ppm, respectively. The HMBC correlation (Figure 2 and Figure S6) from H-5 to C-4 revealed the connection of C-4a to C-4, whereas those from H_2_-9 to C-2, C-11/C-15 indicated that C-9 was attached to C-10 and the remaining *sp*^2^ carbon C-2 (*δ*_C_ 157.7). The NMR data of **1** (Table 1) displayed similar signals to those of 2-(4-hydroxybenzyl)quinazolin-4(3*H*)-one, a quinazolinone isolated from a *Cordyceps*-colonizing fungus *Isaria farinose* [16]. However, an extra methyl signal (*δ*_H_ 1.67 and *δ*_C_ 25.3) and a quaternary carbon (*δ*_C_ 175.8) were present in compound **1**. The key HMBC correlation from H-2′ to C-1′ suggested that compound **1** was the acetyl derivative of 2-(4-hydroxybenzyl)quinazolin-4(3*H*)-one.

**Figure 3:**
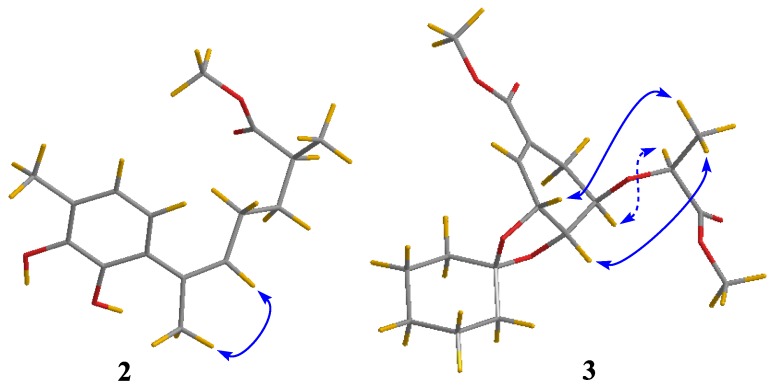
NOESY correlations (blue lines: β-orientation; blue dotted lines: α-orientation) of compounds **2** and **3**.

**Figure 4:**
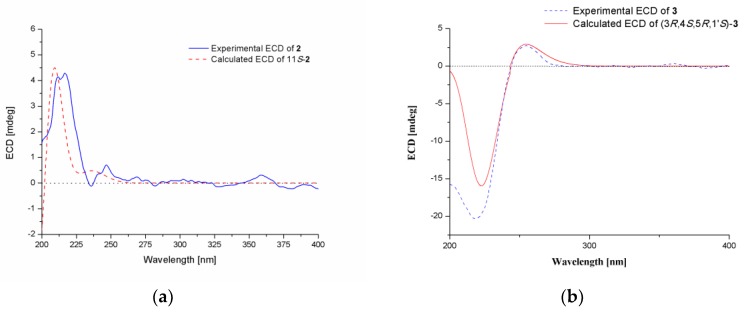
Experimental and calculated ECD spectra of compounds **2** (**a**) and **3** (**b**).

The molecular formula of compound **2** was determined to be C_16_H_22_O_4_ based on its HRESIMS ion at *m/z* 301.1410 [M + Na]^+^ (calculated for C_16_H_22_O_4_Na, 301.1410) (Figure S7), indicating six degrees of unsaturation. The ^1^H and ^13^C-NMR spectroscopic data (Table 1 and Figures S8 and S9) along with HSQC correlations revealed the presence of 16 carbon atoms, which were assigned as six quaternary carbons, four methines including two aromatic methines at δ 119.1 (C-2) and 120.5 (C-3), two methylenes, and four methyls (with one oxygenated at δ 51.1, C-1′). The ^1^H-^1^H COSY correlation (Figure 2 and Figure S10) between H-2 (*δ* 6.28) and H-3 (*δ* 6.49) as well as the HMBC correlations (Figure 2 and Figure S12) between H-2/C-6 and C-4, H-3/C-1 and C-5 revealed a 1,4,5,6-tetrasubstituted aryl ring. The HMBC correlations from H-3 to C-13, and from H-13 to C-3 and C-5 assigned the link of Me-13 and C-4. There should be hydroxyl groups bonded to C-5 and C-6, respectively, according to the downfield shifts of C-5 (*δ* 143.2) and C-6 (*δ* 142.1). The HMBC correlations from H-2 to C-7, and from H-14 to C-1 revealed the connectivity between C-7 and C-1. The HMBC correlations from H-14 to C-8, and from H-8 to C-1 and C-14 assigned the link of Me-14 and C-7. Moreover, this moiety was further extended from C-8 to C-11 and from C-11 to C-15 by ^1^H-^1^H COSY correlations, where C-8 was connected to C-7 on the basis of the HMBC correlations of H-8/C-14 and H-14/C-8. On the other hand, the HMBC correlations from H-10, H-15 to C-12 combined with the downfield chemical shift of C-12 indicated that an ester carbonyl (*δ* 176.1, C-12) was bonded to C-11. The HMBC correlations from H-1′ to C-12 indicated the connectivity between them through an ester bond. Thus, the structure of compound **2** was assigned as a bisabolene-type sesquiterpenoid. The key NOE correlation (Figure 3 and Figure S13) between H-8 and H-14 assigned the *Z*-double bond in C-7/C-8 (7*Z*). The experimental ECD spectra of compound **2** matched well with that calculated for (11*S*)-**2** (Figure 4 and Figure S14), leading to the absolute configuration of compound **2** being determined as 11*S*.

Compound **3** was isolated as colorless oil. HRESIMS ions at *m/z* 355.1744 [M + H]^+^ and 377.1561 [M + Na]^+^ implied that its molecular formula was C_18_H_26_O_7_ (calculated for C_18_H_27_O_7_, 355.1751 and C_18_H_26_O_7_Na, 377.1571) (Figure S15), indicating six degrees of unsaturation. Detailed analysis of the 1D and 2D NMR spectra data (Table 2 and Figures S16 and S17) showed that the signals of **3** were similar to those of methyl[(l*R*),lα,5β,6α]-5-(l-methyl-2-methoxy-2-oxoethyl)-8,8-dimethyl-7,9-dioxabicyclo[4.3.0]non-2-ene-3-carboxylate, a biosynthetically interrelated intermediate which deals with synthesis of 5-enolpyruvylshikimic acid-3-phosphate (EPSP) analogues [17], revealing that compound **3** may also be produced by the same shikimic acid pathway. The main differences between them were the presence of five methylene groups at *δ*_H/C_ 1.46/37.7 (C-2′′), 1.51/24.0 (C-3′′), 1.31/25.0 (C-4′′), 1.48/23.8 (C-5′′), and 1.52/35.4 (C-6′′) in compound **3**. Furthermore, these extra methylenes were linked into a monosubstituted cyclohexyl moiety with one oxygenated quaternary carbon at *δ*_C_ 109.5 (C-1′′), based on the ^1^H-^1^H COSY correlations (Figure 2 and Figure S18) between H-2′′/H-3′′, H-3′′/H-4′′, H-4′′/H-5′′ and H-5′′/H-6′′, and the key HMBC correlations (Figure 2 and Figure S20) from H-3′′ and H-5′′ to C-1′′. Combined with the key HMBC correlations from H-3 and H-4 to C-1′′, the structure of compound **3** was definitively established.

The relative configuration of compound **3** was assigned by the analysis of NOESY data (Figure 3 and Figure S21). The key NOE correlations of H-3′ with H-3 and H-4 indicated that they were on the same side of the molecule, while the NOE correlation between H-5 and H-1′ revealed them on the other face. On the basis of the above evidence, the relative configuration of compound **3** was determined. The absolute configuration of compound **3** was studied by TDDFT-ECD calculations. The ECD spectrum of compound **3** exhibited negative Cotton Effect (CE) at 221 nm and positive CE at 250 nm, which matched well with that calculated for (3*R*,4*S*,5*R*,1′*S*)-**3** (Figure 4 and Figure S22).

In addition to the new compounds **1**–**3**, one known quinazolinone alkaloid, 2-(4-hydroxybenzoyl)-4(3*H*)-quinazolinone (**4**) [18], and one known triazole derivative, chrysotriazoles A (**5**) [19], were also isolated and identified from *A. sydowii* SW9.

The obtained compounds **1**–**5** were tested for antimicrobial activities against four human pathogenic bacterial strains (Table 3). The new compound **1** exhibited obvious inhibitory activity against *S. epidermidis*, with an MIC value of 4.0 μg/mL. Compound **2** showed significant inhibitory activity on *E*. *coli*, with an MIC value of 2.0 μg/mL, comparable to that of the positive control chloramphenicol (MIC 2.0 μg/mL). Moreover, compound **2** also exhibited potent activity against *S. pneumoniae*, with an MIC value of 4.0 μg/mL.

## 3. Materials and Methods

### 3.1. General Experimental Procedures

Optical rotations were measured on an Optical Activity AA-55 polarimeter (Optical Activity Ltd., Cambridge, UK). UV spectra were recorded on a Lengguang Gold Spectrumlab-54 spectrophotometer (Shanghai Lengguang Technology Co. Ltd., Shanghai, China). ECD spectra were obtained on a Chirascan spectropolarimeter (Applied Photophysics Ltd., Surrey, UK). 1D and 2D NMR spectra were recorded at 500 and 125 MHz for ^1^H and ^13^C, respectively, on a Bruker Avance 500 MHz spectrometer (Bruker Corp., Billerica, MA, USA). High-resolution ESI mass spectra were determined on an Agilent G6230 TOF mass spectrometer (Agilent Technologies Inc., Santa Clara, CA, USA). Column chromatography was performed with silica gel (200−300 mesh, Qingdao Haiyang Chemical Co., Qingdao, China), RP-18 (AAG12S50, YMC Co., Ltd., Kyoto, Japan), and Sephadex LH-20 (GE Healthcare, Uppsala, Sweden). The solvents (CHCl_3_, EtOH, EtOAc, MeOH, and PE) were of analytical grade and distilled prior to use. Quantum chemical calculations were run with Gaussian 09 software (Gaussian, Inc., Wallingford, CT, USA).

### 3.2. Fungal Material

The fungus *Aspergillus sydowii* SW9 was isolated from a seawater sample collected in August 2016, from Yangma Island, Yantai, China. The fungus was identified by morphological observation and analysis of the Internal Transcribed Spacer (ITS) regions of its rDNA, whose sequence data were deposited at GenBank with the accession number MN696205.

### 3.3. Fermentation

Fungal fermentation was carried out statically at room temperature for 35 days in 80 × 1 L Erlenmeyer flasks, each containing 20% potato juice, 2% glucose, 0.5% peptone, and 0.3% yeast extract.

### 3.4. Extraction and Isolation

The mycelia were separated from the culture broth by filtration, and they were dried in the shade and exhaustively extracted with a mixture of ethyl alcohol and H_2_O (95:5, *v*/*v*). After removing organic solvents by evaporation under vacuum, the residue was partitioned between EtOAc and H_2_O to produce an EtOAc soluble extract. The filtrate was directly extracted with EtOAc and then concentrated to afford an extract. Since the TLC and HPLC profiles of the two EtOAc extracts were almost identical, they were combined and concentrated under reduced pressure to produce an extract (27.2 g) for further separation.

The organic extract was fractionated by vacuum liquid chromatography (VLC) on silica gel, eluting with different solvents of increasing polarity from petroleum ether (PE) to MeOH to yield nine fractions (Frs. 1–9), which were pooled based on TLC analysis. Fr. 4 (4.5 g), eluted with PE–EtOAc (2:1), was further purified by column chromatography (CC) on Sephadex LH-20 (MeOH) to afford **4** (15.6 mg) and **5** (13.9 mg). Fr. 6 (2.1 g), eluted with CHCl_3_–MeOH (10:1), was purified by CC on silica gel eluting with a CHCl_3_–MeOH gradient (40:1 to 10:1) to afford two subfractions (Fr. 6-1 and Fr. 6-2). Fr. 6-1 was further purified by CC over RP-18 eluting with a MeOH–H_2_O gradient (1:9 to 1:0) to obtain **1** (15.4 mg). Fr. 6-2 was further purified by Sephadex LH-20 (MeOH) to yield **3** (15.7 mg). Fr. 7 (3.7 g), eluted with CHCl_3_–MeOH (5:1), was further purified by CC on Sephadex LH-20 (MeOH) and then purified by CC over RP-18 eluting with a MeOH–H_2_O gradient (1:9 to 1:0) to obtain **2** (8.5 mg).

*2-(4-Hydroxybenzyl)-4-(3-acetyl)quinazolin-one* (**1**). White powder; UV (MeOH) *λ*_max_ (log *ε*): 204 (2.78), 220 (2.86), 241 (2.83) nm. ^1^H and ^13^C-NMR: see Table 1. HRESIMS: *m/z* 295.1079 [M + H]^+^ (calculated for C_17_H_15_N_2_O_3_, 295.1077).

*Methyl(R,E)-6-(2,3-dihydroxy-4-methylpenyl)-2-methylhept-5-enoate* (**2**): Colorless oil; [α${]}_{D}^{20}$ = −2.1 (*c* 0.4, MeOH); UV (MeOH) *λ*_max_ (log *ε*) 205 (1.80) nm; ECD (0.45 mg/mL, MeOH) *λ*_max_ (Δ*ε*) 215 (+3.20) nm; ^1^H and ^13^C-NMR data, Table 1; HRESIMS *m/z* 301.1410 [M + Na]^+^ (calculated for C_16_H_22_O_4_Na, 301.1410).

*Sydowether* (**3**): Colorless oil; [α${]}_{D}^{20}$ = +1.0 (*c* 0.45, MeOH); UV (MeOH) *λ*_max_ (log *ε*) 201 (1.05) nm; ECD (0.30 mg/mL, MeOH) *λ*_max_ (Δ*ε*) 221 (−8.13), 250 (+0.15) nm; ^1^H and ^13^C-NMR data, Table 2; HRESIMS *m/z* 355.1744 [M + H]^+^ (calculated for C_18_H_27_O_7_, 355.1751), 377.1561 [M + Na]^+^ (calculated for C_18_H_26_O_7_Na, 377.1571).

### 3.5. Antimicrobial Assays

Antimicrobial evaluation against pathogenic bacteria (*Escherichia coli*, *Staphylococcus aureus*, *Staphylococcus epidermidis,* and *Streptococcus pneumoniae*) was assayed as described previously [20], with chloramphenicol as the positive control. The specific experiment was as follows: the initial cultures were maintained on Luria Broth (LB) agar plates for each pathogenic strain, picking bacterial colonies, and suspended in Mueller–Hinton broth to approximately 5 × 10^5^ CFU/mL. Aliquots of bacterial suspension (95 μL) and compound dilution (5 μL) were then added to each well (to give final compound concentrations of 128 to 0.125 μg/mL in 2.0% DMSO), and the plate was incubated at 37 °C aerobically for 24 h. After that, the optical density of each well at 600 nm was measured using a Tecan GENios multifunctional microplate reader (infinite M1000 PRO, Männedorf, Switzerland). Then, MIC values were defined as the minimum concentration of compound that inhibited visible bacterial growth. The pathogenic bacterial strains were provided by the Key Laboratory of Marine Biotechnology in Universities of Shandong, Ludong University.

### 3.6. Computational Section

The conformational searches of compounds **2** and **3** were performed via molecular mechanics using the MM+ method in HyperChem software (Version 8.0, Hypercube, Inc., Gainesville, FL, USA), and the geometries were further optimized at the B3LYP/6-31G(d) level via Gaussian 09 software (Version D.01; Gaussian, Inc., Wallingford, CT, USA) [21] to give the energy-minimized conformers. Then, the optimized conformers were subjected to the calculation of ECD by using TDDFT at B3LYP/6-31G level. Solvent effects of the MeOH solution were evaluated at the same DFT level using the SCRF/PCM method.

## 4. Conclusions

One new alkaloid, 2-(4-hydroxybenzyl)-4-(3-acetyl)quinazolin-one (**1**), one new aromatic bisabolene-type sesquiterpenoid (**2**) and one new chorismic acid analogue (**3**), along with two known quinazolinone alkaloids (**4**, **5**), were isolated and identified from the culture extract of *Aspergillus sydowii* SW9, a seawater-derived fungus. The structures of the new compounds were elucidated using NMR and HRESIMS data analysis as well as quantum chemical ECD calculations. All of the isolated compounds were tested for antimicrobial activities against four human pathogenic bacterial strains. The new compounds **1** and **2**, and the known compound **4** exhibited obvious inhibitory activities against the human pathogenic bacterial strains tested.

## Figures and Tables

**Table 1:** ^1^H and ^13^C data of compounds **1** and **2** (measured in DMSO-*d*_6_).

	1			2	
No.	*δ*_H_ (Mult, *J* in Hz) ^a^	*δ*_C_, Type ^b^	No.	*δ*_H_ (Mult, *J* in Hz) ^a^	*δ*_C_, Type ^b^
1	-	-	1	-	126.6, qC
2	-	157.7, qC	2	-	119.1, CH
3	-	-	3	6.28, d (7.6)	120.5, CH
4	-	162.9, qC	4	6.49, d (7.6)	123.2, qC
4a	-	121.1, qC	5	-	143.2, qC
5	8.04, d (8.0)	126.1, CH	6	-	142.1, qC
6	7.42, t (7.5)	126.3, CH	7	-	134.2, qC
7	7.73, t (7.5)	134.5, CH	8	5.34, t (6.6)	126.7, CH
8	7.57, d (8.0)	127.1, CH	9	1.74, m	26.3, CH_2_
8a	-	149.5, qC	10a	1.54, m	33.1, CH_2_
9	3.79, s	40.7, CH_2_	10b	1.33, m	-
10	-	127.0, qC	11	2.33, m	38.0, CH
11	7.14, d (7.8)	130.2, CH	12	-	176.1, qC
12	6.70, d (7.8)	115.7, CH	13	0.94, d (6.6)	16.1, CH_3_
13	-	157.0, qC	14	1.88, s	24.6, CH_3_
14	6.70, d (7.8)	115.7, CH	15	2.10, s	16.5, CH_3_
15	7.14, d (7.8)	130.2, CH	1′	3.50, s	51.1, CH_3_
1′	-	175.8, qC			
2′	1.67, s	25.3, CH_3_			

^a^ Measured at 500 MHz; ^b^ Measured at 125 MHz.

**Table 2:** ^1^H and ^13^C data of compound **3** (measured in DMSO-*d*_6_).

No.	*δ*_H_ (Mult, *J* in Hz) ^a^	*δ*_C_, Type ^b^	No.	*δ*_H_ (Mult, *J* in Hz) ^a^	*δ*_C_, Type ^b^
1	-	129.4, qC	1′′	-	109.5, qC
2	6.71, s	135.0, CH	2′′	1.46, m	37.7, CH_2_
3	4.71, s	71.7, CH	3′′	1.51, m	24.0, CH_2_
4	4.16, t (6.2)	75.1, CH	4′′	1.31, m	25.0, CH_2_
5	3.72, m	75.5, CH	5′′	1.48, m	23.8, CH_2_
6a	2.26, dd (6.5, 17.4)	26.8, CH_2_	6′′	1.52, m	35.4, CH_2_
6b	2.46, dd (6.5, 17.4)				
7	-	166.6, qC	1′′′	3.63, s	52.0, CH_3_
1′	4.23, m	73.8, CH	1′′′′	3.68, s	52.5, CH_3_
2′	-	173.6, qC			
3′	1.22, d (6.8)	19.5, CH_3_			

^a^ Measured at 500 MHz; ^b^ Measured at 125 MHz.

**Table 3:** Antimicrobial activities of compounds **1**–**5** (MIC, μg/mL) ^a^.

Strains	1	2	3	4	5	Positive Control
*Escherichia coli* ^b^	16	2.0	–	8.0	–	2.0
*Staphylococcus aureus* ^b^	8.0	16	–	8.0	32	1.0
*Staphylococcus. epidermidis* ^b^	4.0	16	32	16	–	2.0
*Streptococcus pneumoniae* ^b^	16	4.0	32	8.0	32	1.0

^a^ (–) = MIC > 32 μg/mL, ^b^ Chloramphenicol as positive control.

## Supplementary Materials

The following are available online at https://www.mdpi.com/1420-3049/24/24/4596/s1, HRESIMS, 1D and 2D NMR spectra, UV spectra and ECD spectra of compounds **1**–**3**.
