# Supplementary material for: Antimicrobial Secondary Metabolites from the Seawater-Derived Fungus Aspergillus sydowii SW9
Source: Molecules. 2019 Dec 16;24(24):4596. doi: 10.3390/molecules24244596 (PMC6943586; doi:10.3390/molecules24244596)

Supplementary File

for

Antimicrobial Secondary Metabolites from the Seawater-Derived Fungus *Aspergillus sydowii* SW9

Yu-Jing Liu [†,1], Jian-Long Zhang [†,1,3], Chen Li [1], Xue-Gen Mu [1], Xiao-Li Liu [1], Lei Wang [1], Yan-Cui Zhao [1], Peng Zhang [4], Xiao-Dong Li [1,2,*] and Xing-Xiao Zhang [1,*]

[1] Key Laboratory of marine biotechnology in Universities of Shandong (Ludong University), School of Life Sciences, Ludong University, Yantai 264025, China; chunxiao6@163.com (Y.-J.L.); Zhangjianlong@ldu.edu.cn (J.-L.Z.); Lychees6601@163.com (C.L.); mxgdmn@163.com (X.-G.M.); lxlshz2006@163.com (X.-L.L.); wanglei9909@163.com (L.W.); yancuizh@aliyun.com (Y.-C.Z.)

[2] Yantai Institute of Coastal Zone Research, Chinese Academy of Sciences, Yantai 264003, China

[3] Shandong Provincial Key Laboratory of Quality Safty Monitoring and Risk Assessment for Animal Products, Ji'nan 250022, China

[4] Tobacco Research Institute, Chinese Academy of Agricultural Sciences, Qingdao 266101, China; zhangpeng@caas.cn (P.Z.)

* Correspondence: imnli@163.com (X.-D.L.); zhangxingxiao@ldu.edu.cn (X.-X.Z.); Tel.: +86-532-8289-8890 (X.-D.L.)

† These authors contributed equally to this work.

Content

Figure S1. HRESI mass spectrum of compound **1**.

Figure S2. ^{1}H NMR (500 MHz, DMSO-d_6) spectrum of compound **1**.

Figure S3. ^{13}C NMR (125 MHz, DMSO-d_6) and DEPT spectra of compound **1**.

Figure S4. COSY (DMSO-d_6) spectrum of compound **1**.

Figure S5. HSQC (DMSO-d_6) spectrum of compound **1**.

Figure S6. HMBC (DMSO-d_6) spectrum of compound **1**.

Figure S7. HRESI mass spectrum of compound **2**.

Figure S8. ^{1}H NMR (500 MHz, DMSO-d_6) spectrum of compound **2**.

Figure S9. ^{13}C NMR (125 MHz, DMSO-d_6) and DEPT spectra of compound **2**.

Figure S10. COSY (DMSO-d_6) spectrum of compound **2**.

Figure S11. HSQC (DMSO-d_6) spectrum of compound **2**.

Figure S12. HMBC (DMSO-d_6) spectrum of compound **2**.

Figure S13. NOESY (DMSO-d_6) spectrum of compound **2**.

Figure S14. ECD and UV spectra of compound **2**.

Figure S15. HRESI mass spectrum of compound **3**.

Figure S16. ^{1}H NMR (500 MHz, DMSO-d_6) spectrum of compound **3**.

Figure S17. ^{13}C NMR (125 MHz, DMSO-d_6) and DEPT spectra of compound **3**.

Figure S18. COSY (DMSO-d_6) spectrum of compound **3**.

Figure S19. HSQC (DMSO-d_6) spectrum of compound **3**.

Figure S20. HMBC (DMSO-d_6) spectrum of compound **3**.

Figure S21. NOESY (DMSO-d_6) spectrum of compound **3**.

Figure S22. ECD and UV spectra of compound **3**.

Figure S23. ^{1}H NMR (500 MHz, CD_3OD) spectrum of compound **4**.

Figure S24. ^{13}C NMR (125 MHz, CD_3OD) spectrum of compound **4**.

Figure S25. ^{1}H NMR (500 MHz, DMSO-d_6) spectrum of compound **5**.

Figure S26. ^{13}C NMR (125 MHz, DMSO-d_6) spectrum of compound **5**.

Figure S1. HRESI mass spectrum of compound **1**.

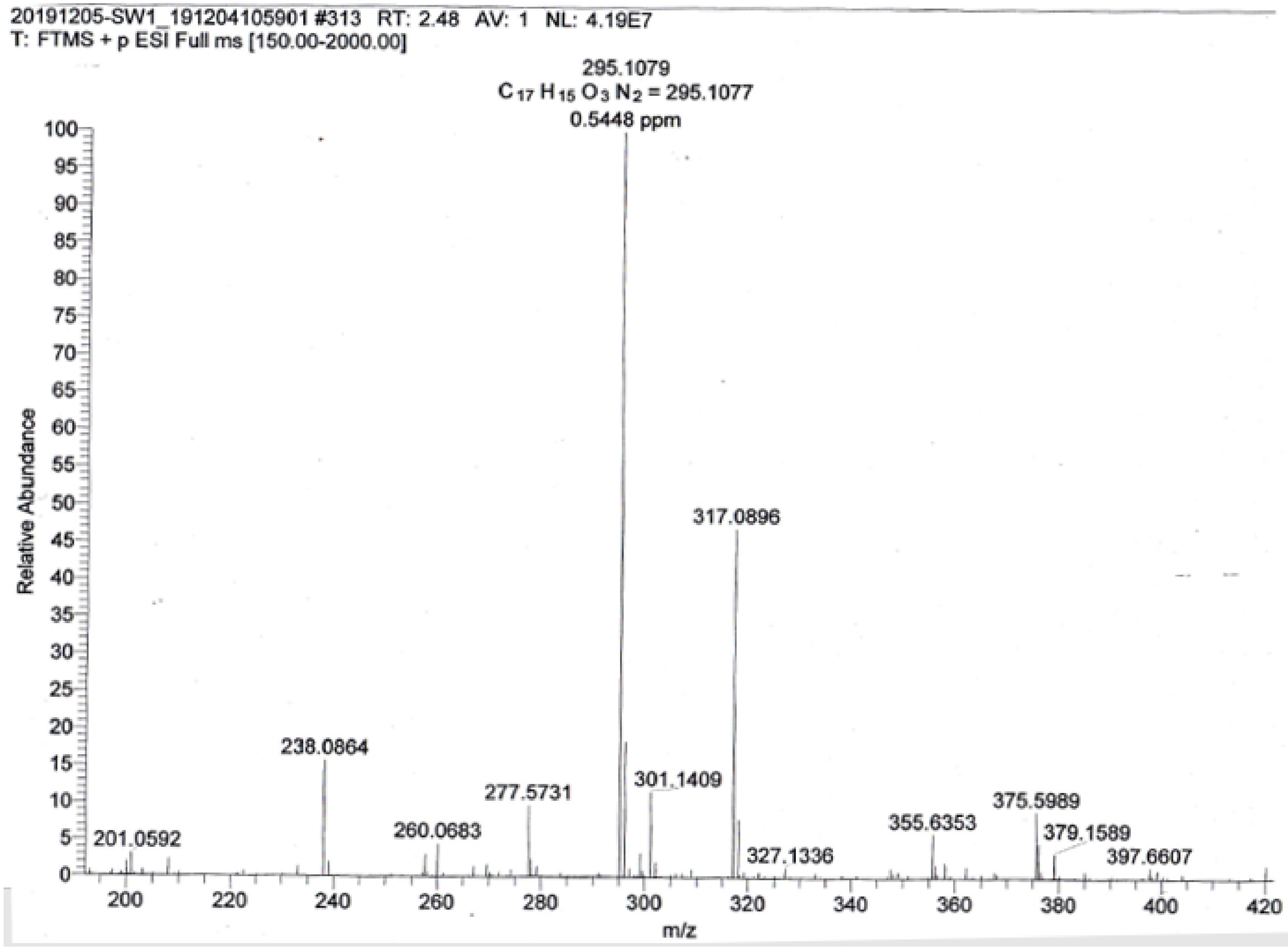

Figure S2. ^{1}H NMR (500 MHz, DMSO-d_6) spectrum of compound **1**.

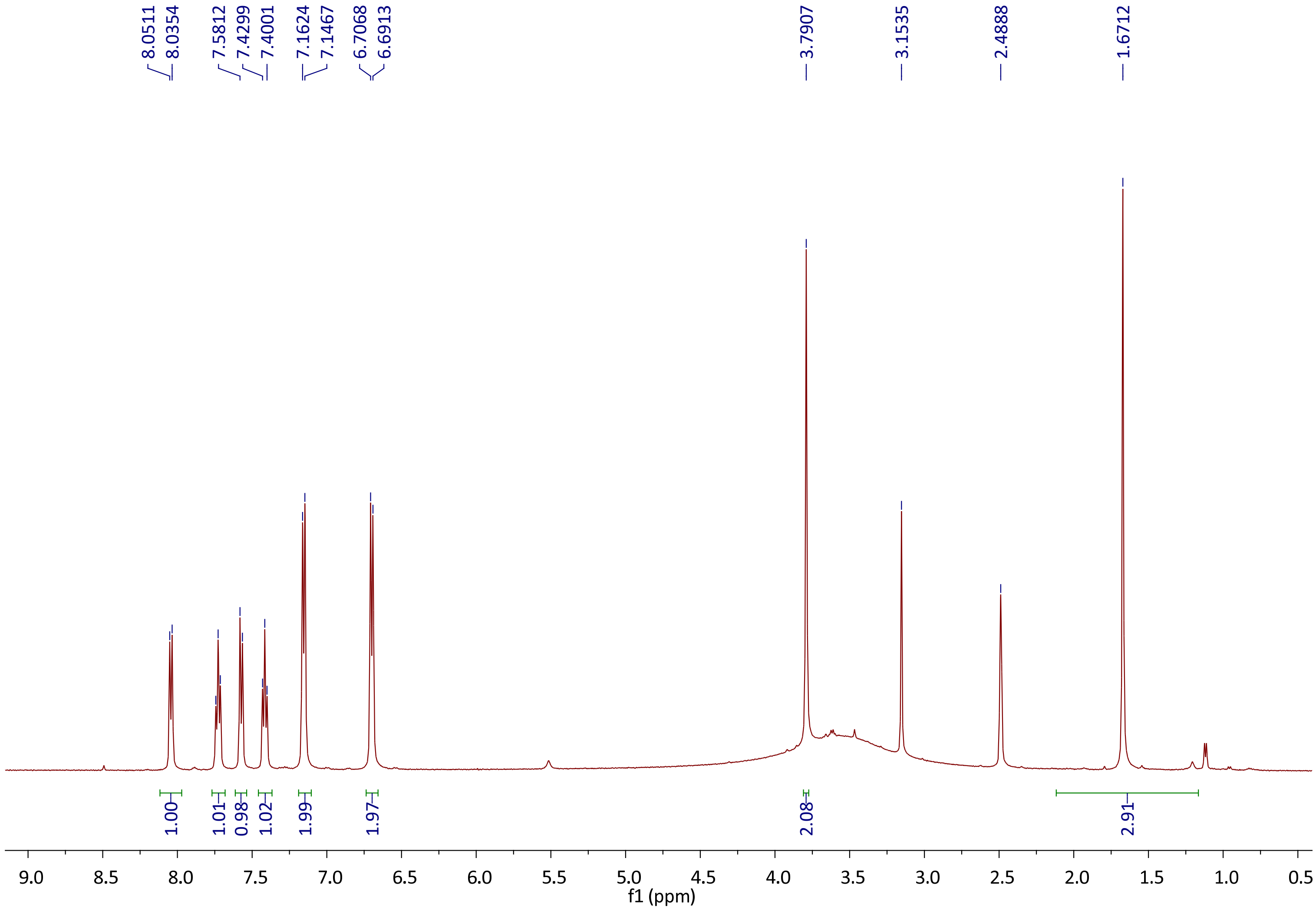

Figure S3. ^{13}C NMR (125 MHz, DMSO-d_6) and DEPT spectra of compound **1**.

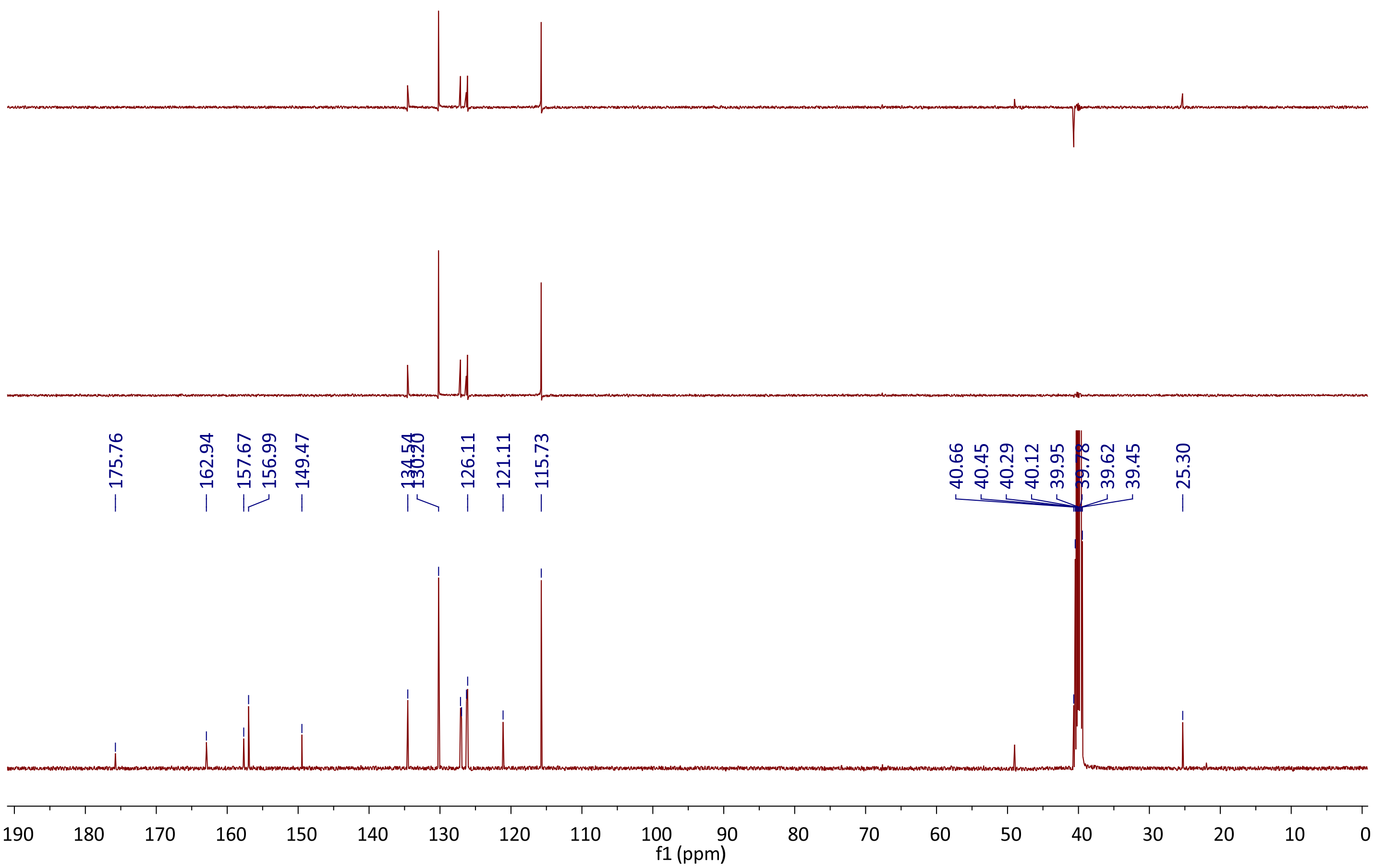

Figure S4. COSY (DMSO-d_6) spectrum of compound **1**.

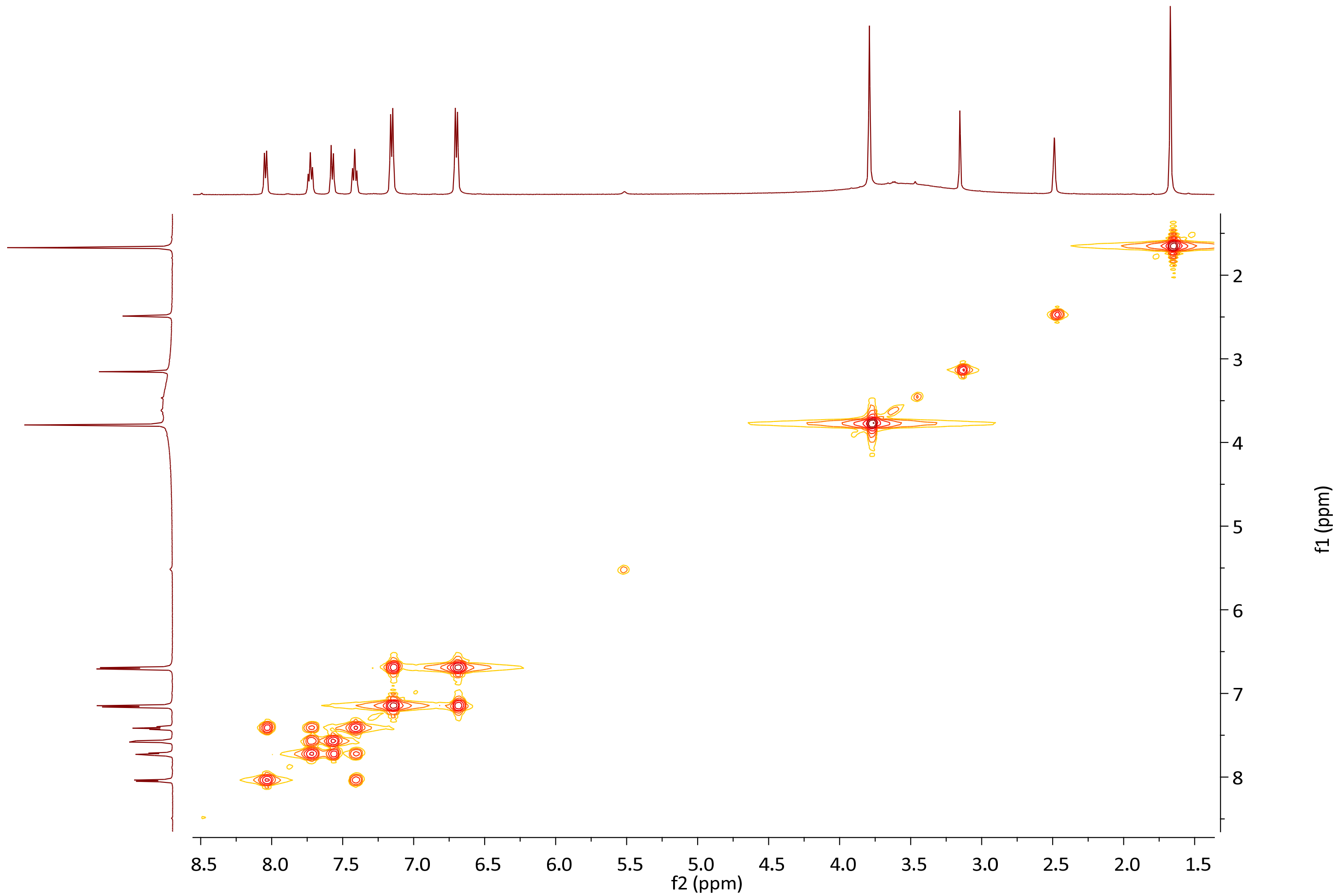

Figure S5. HSQC (DMSO-d_6) spectrum of compound **1**.

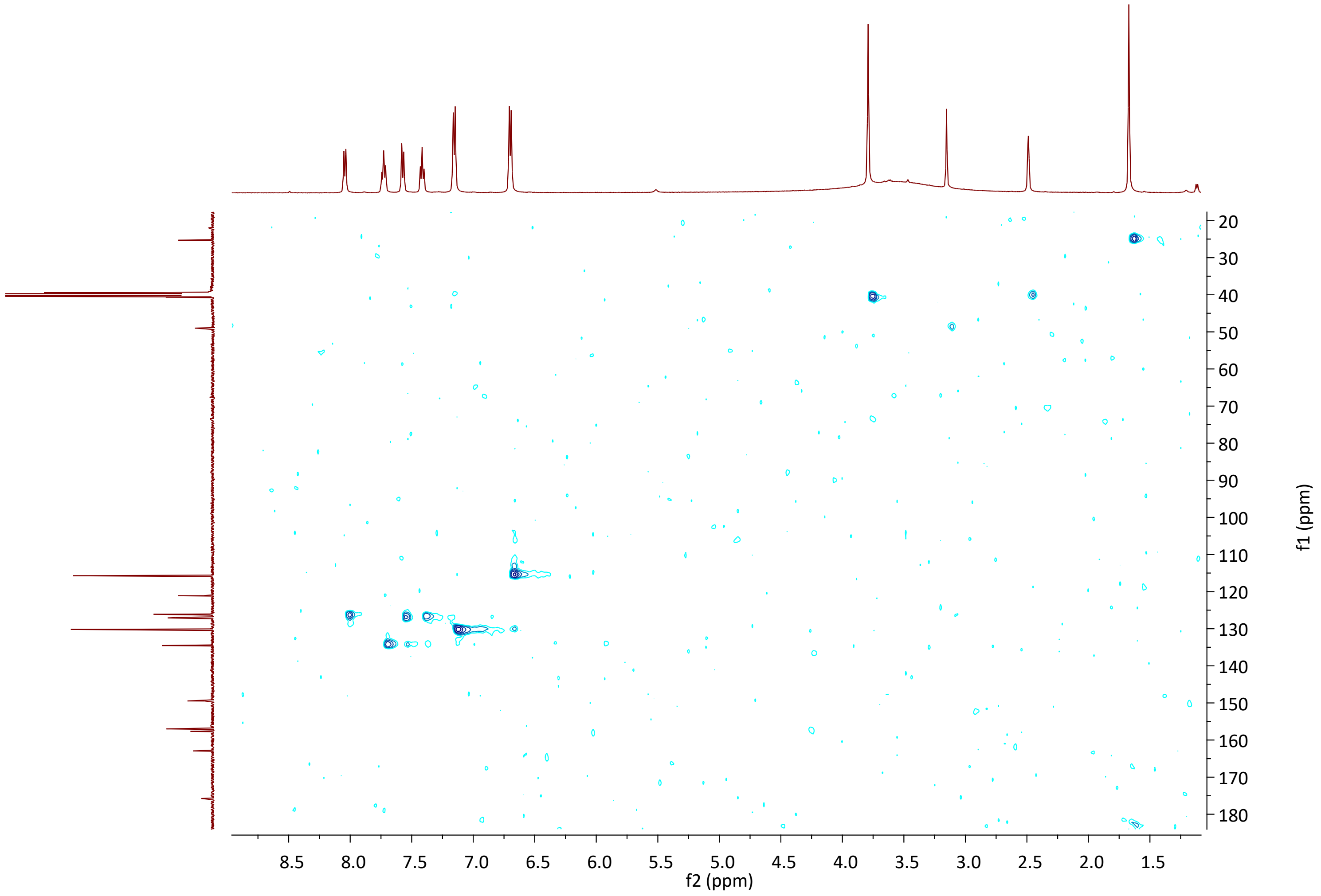

Figure S6. HMBC (DMSO-d_6) spectrum of compound **1**.

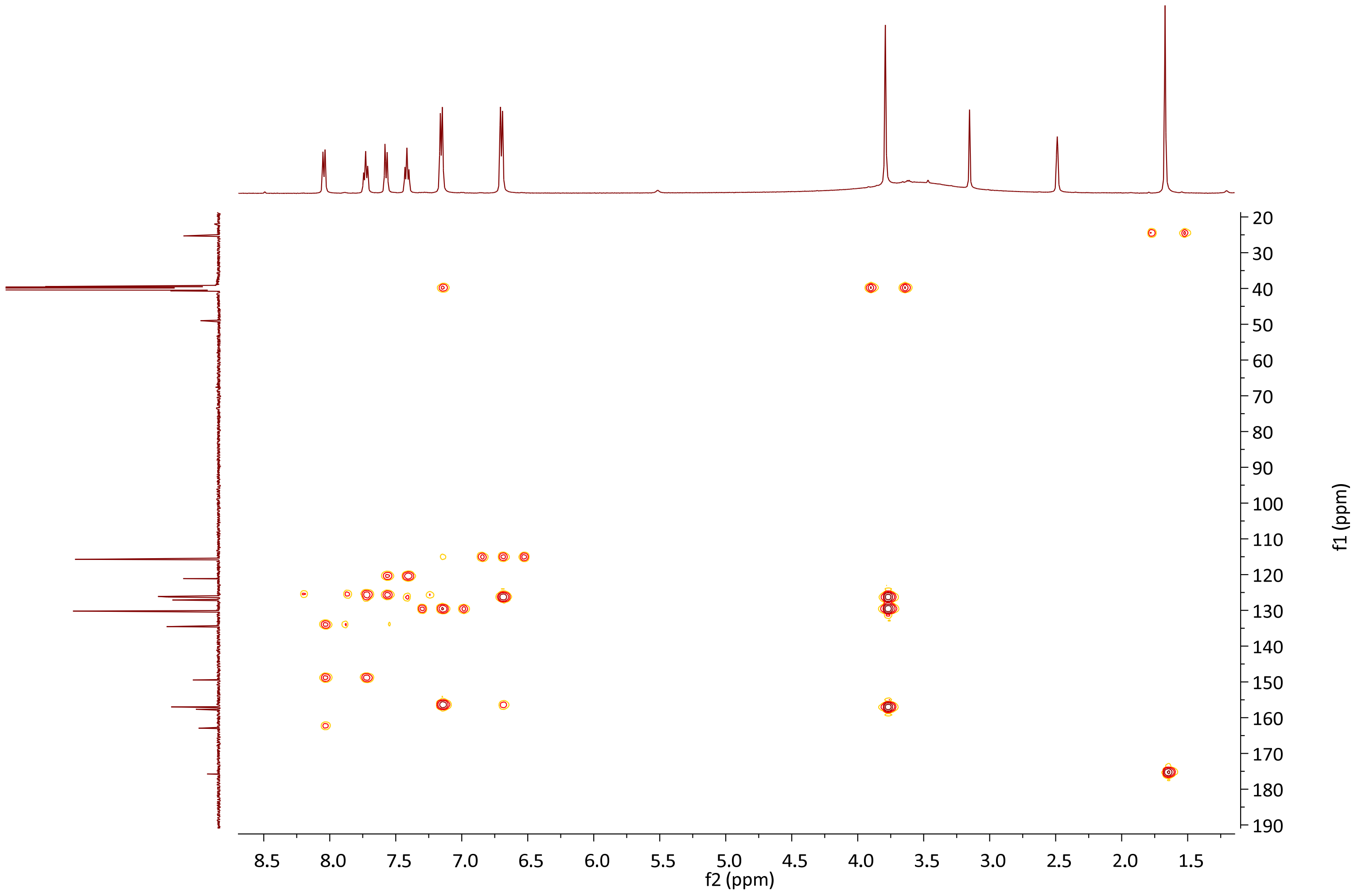

Figure S7. HRESI mass spectrum of compound **2**.

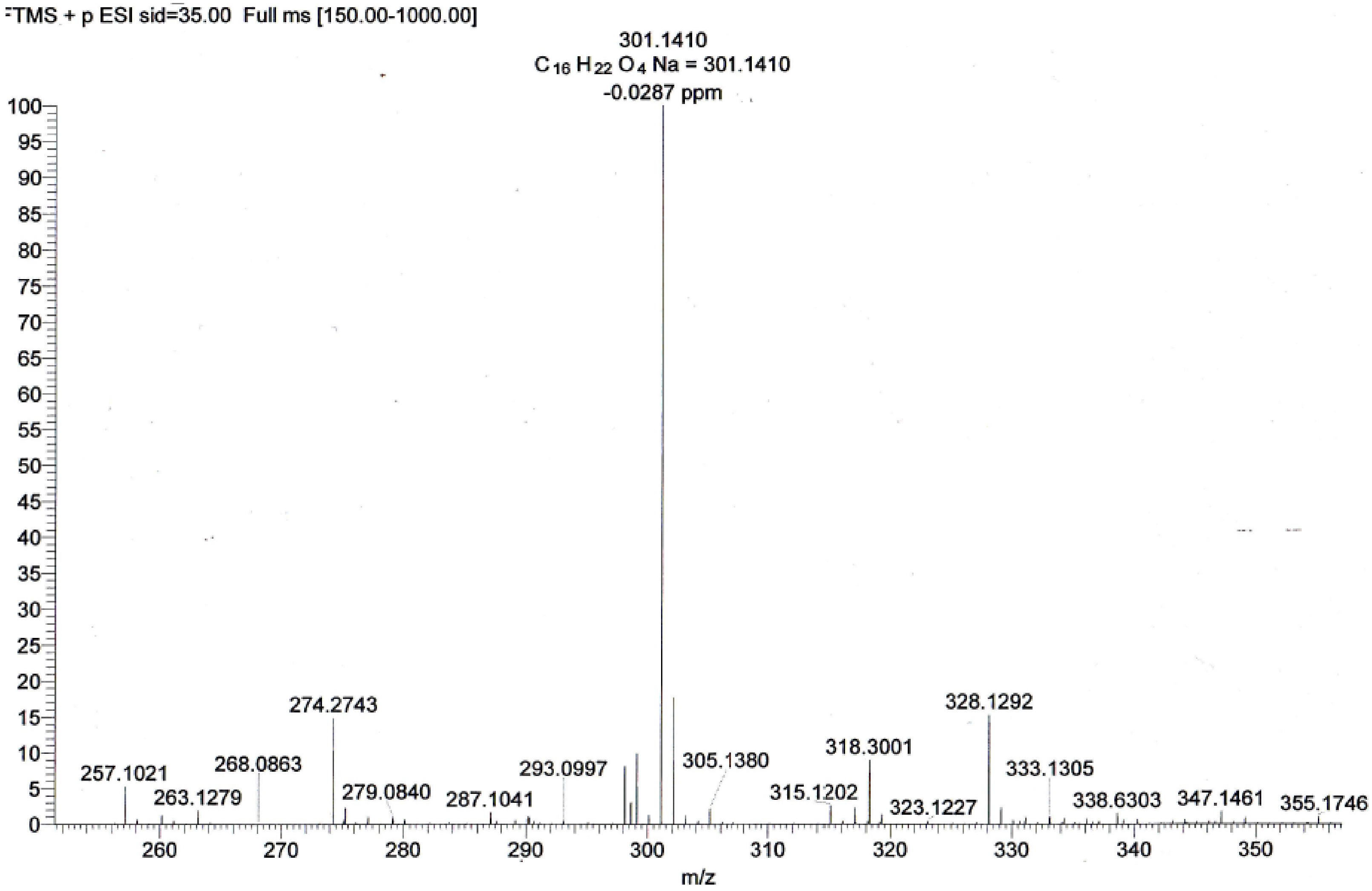

Figure S8. ^{1}H NMR (500 MHz, DMSO-d_6) spectrum of compound **2**.

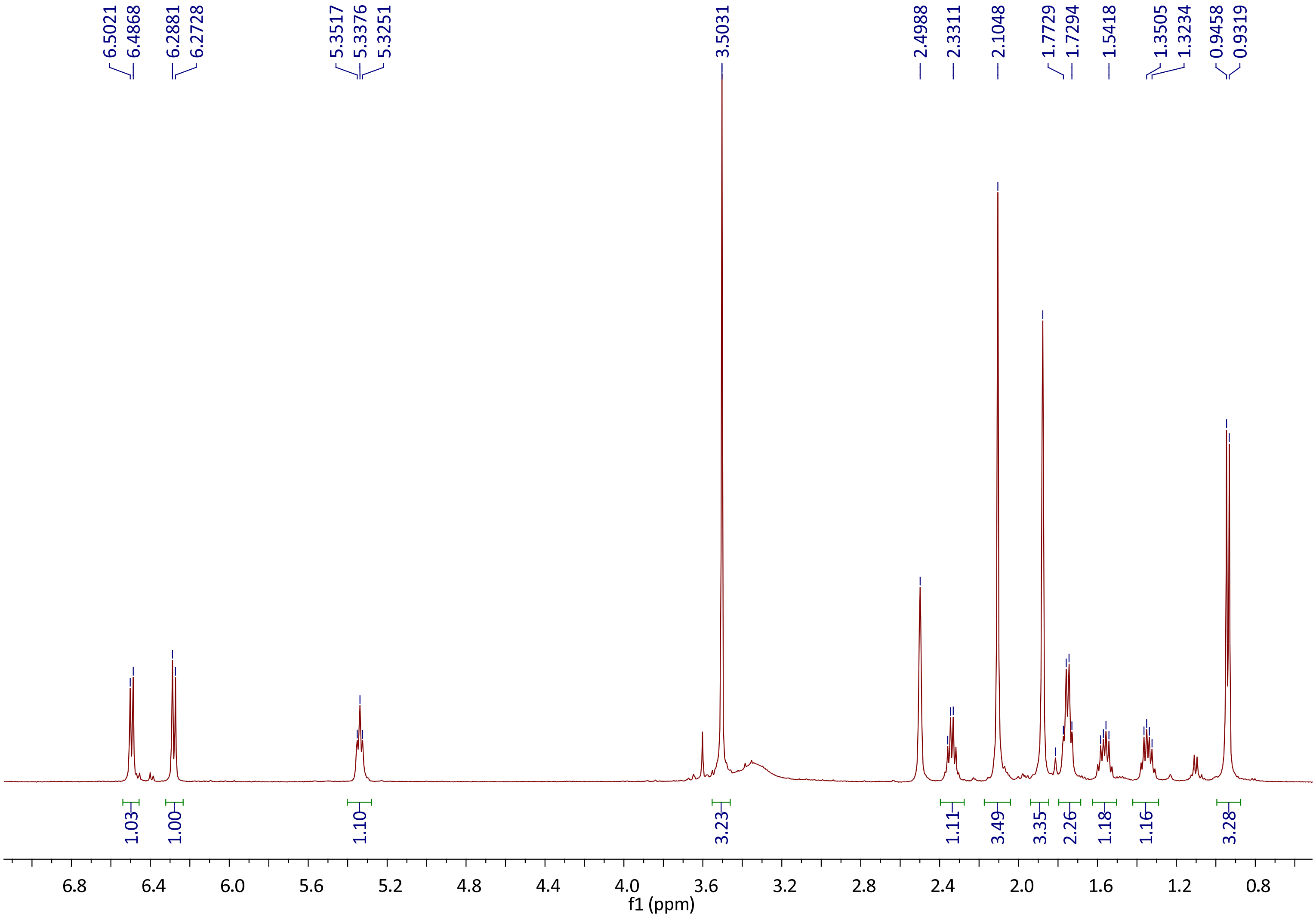

Figure S9. ^{13}C NMR (125 MHz, DMSO-d_6) and DEPT spectra of compound **2**.

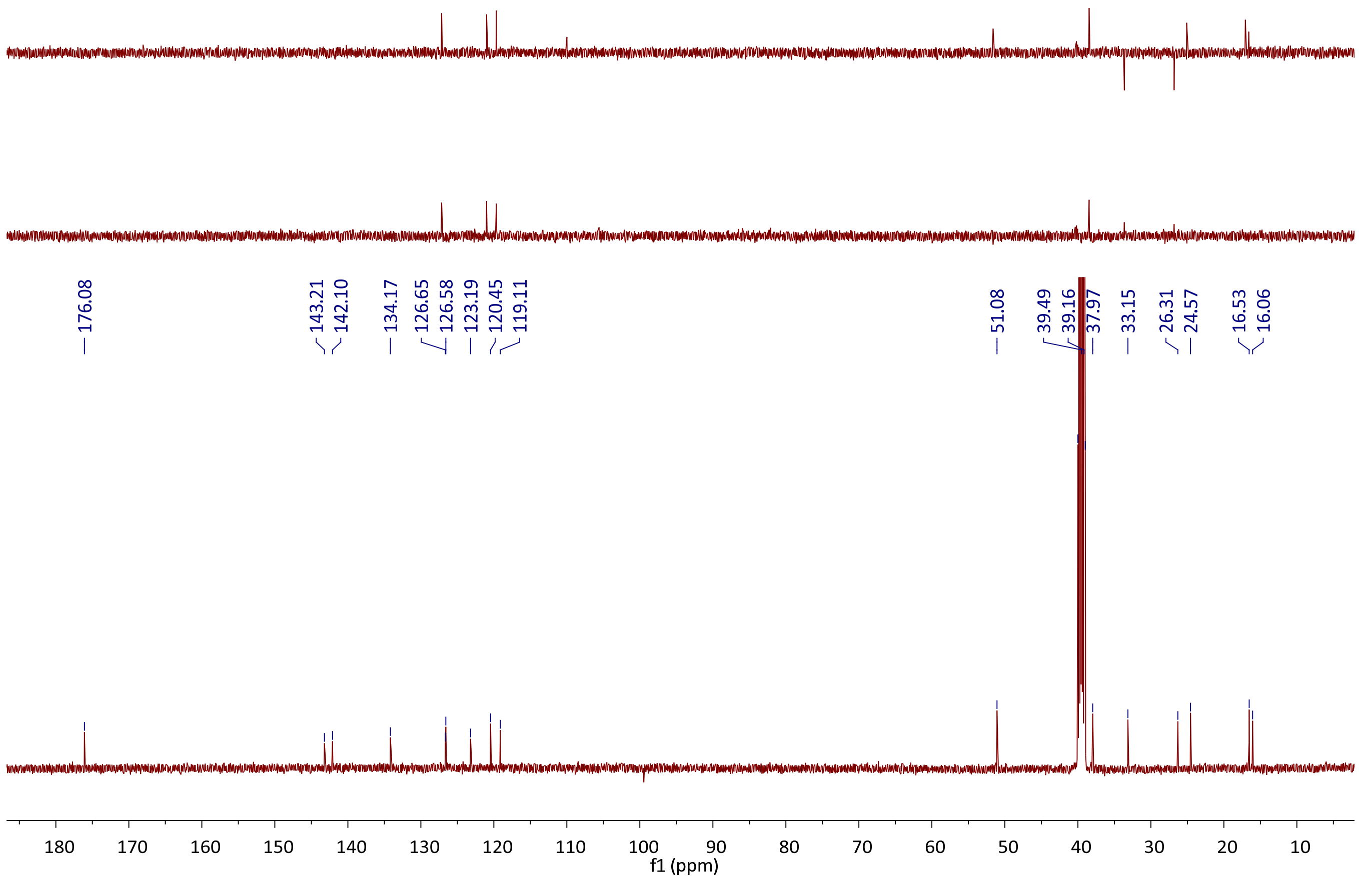

Figure S10. COSY (DMSO-d_6) spectrum of compound **2**.

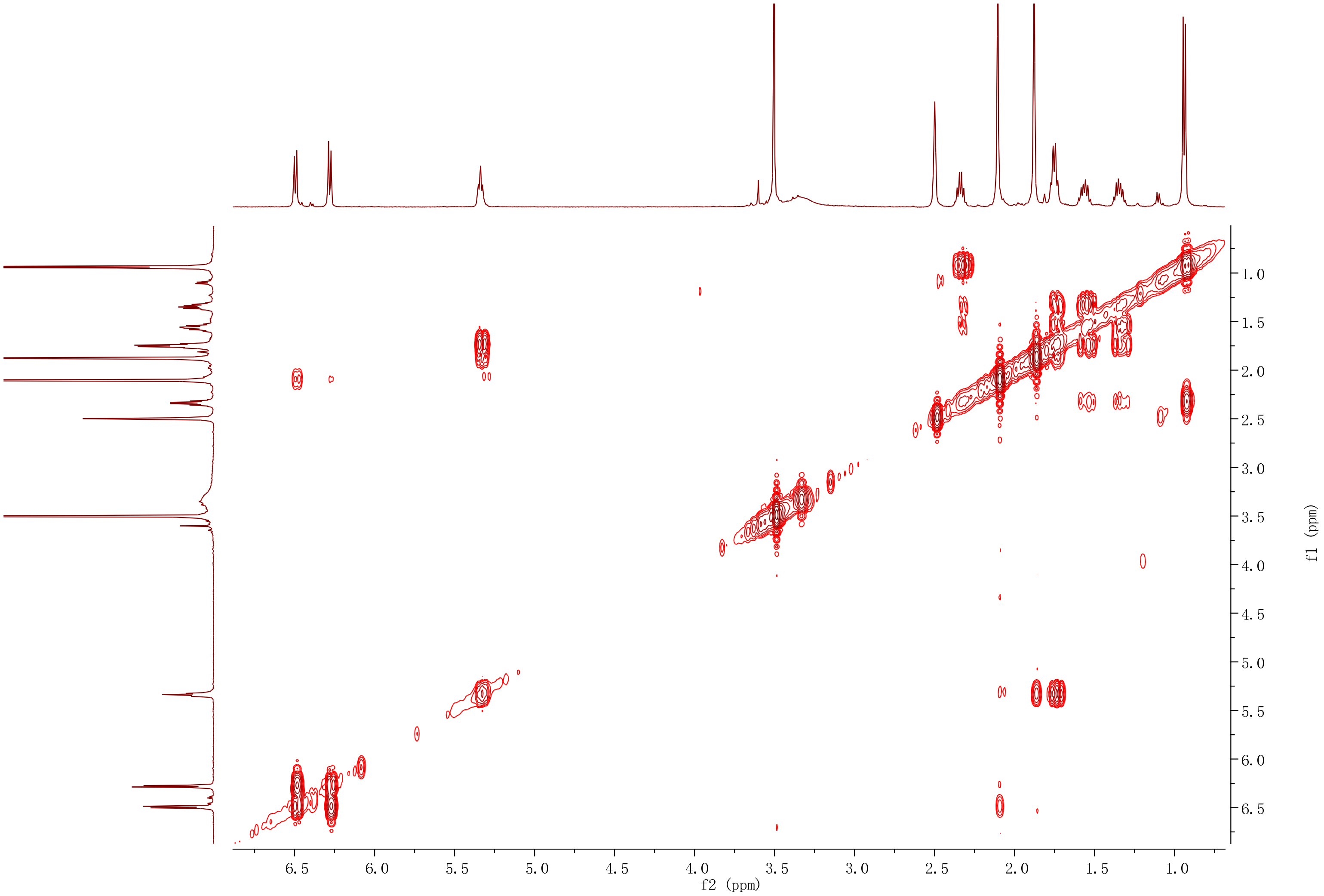

Figure S11. HSQC (DMSO-d_6) spectrum of compound **2**.

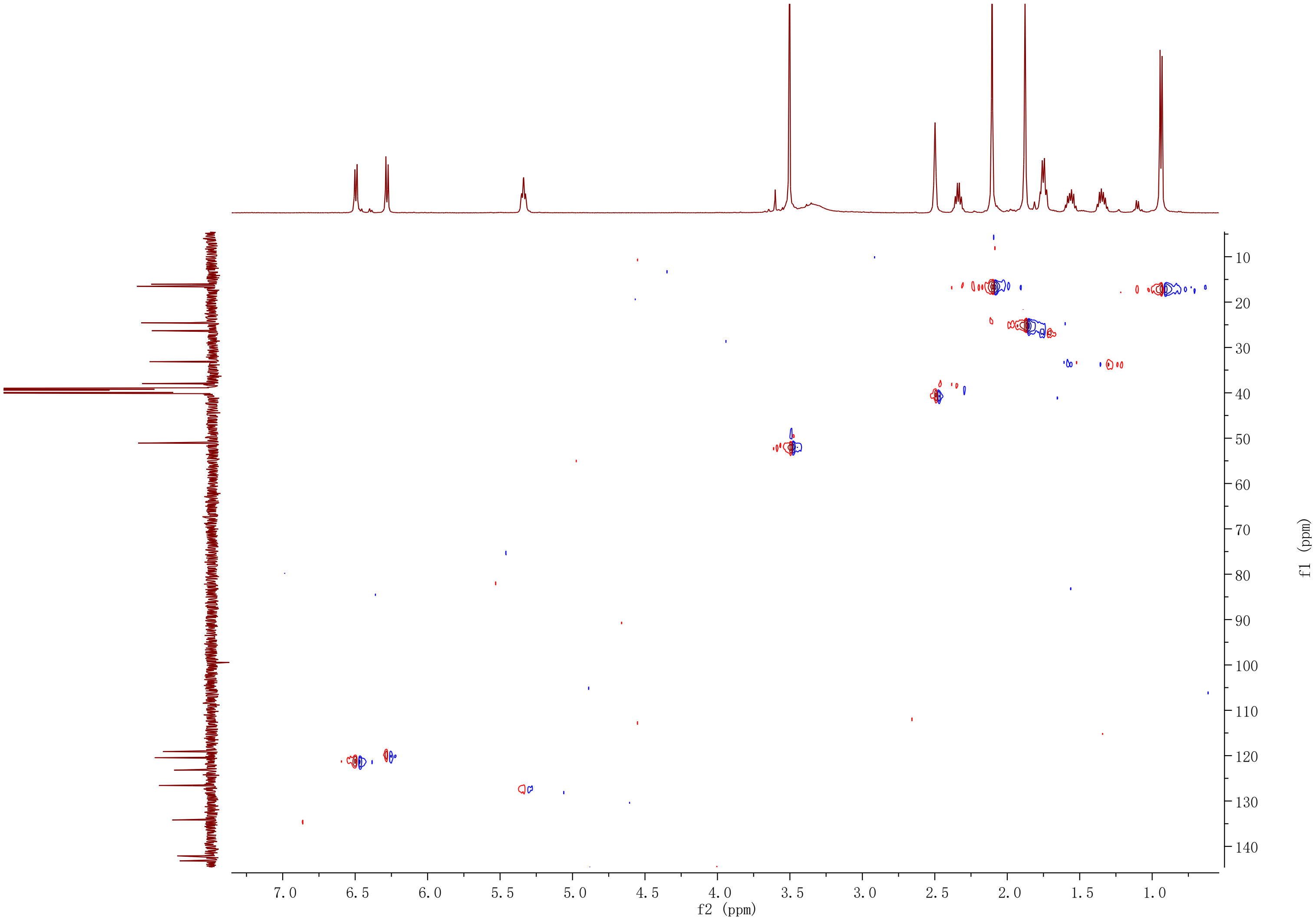

Figure S12. HMBC (DMSO-d_6) spectrum of compound **2**.

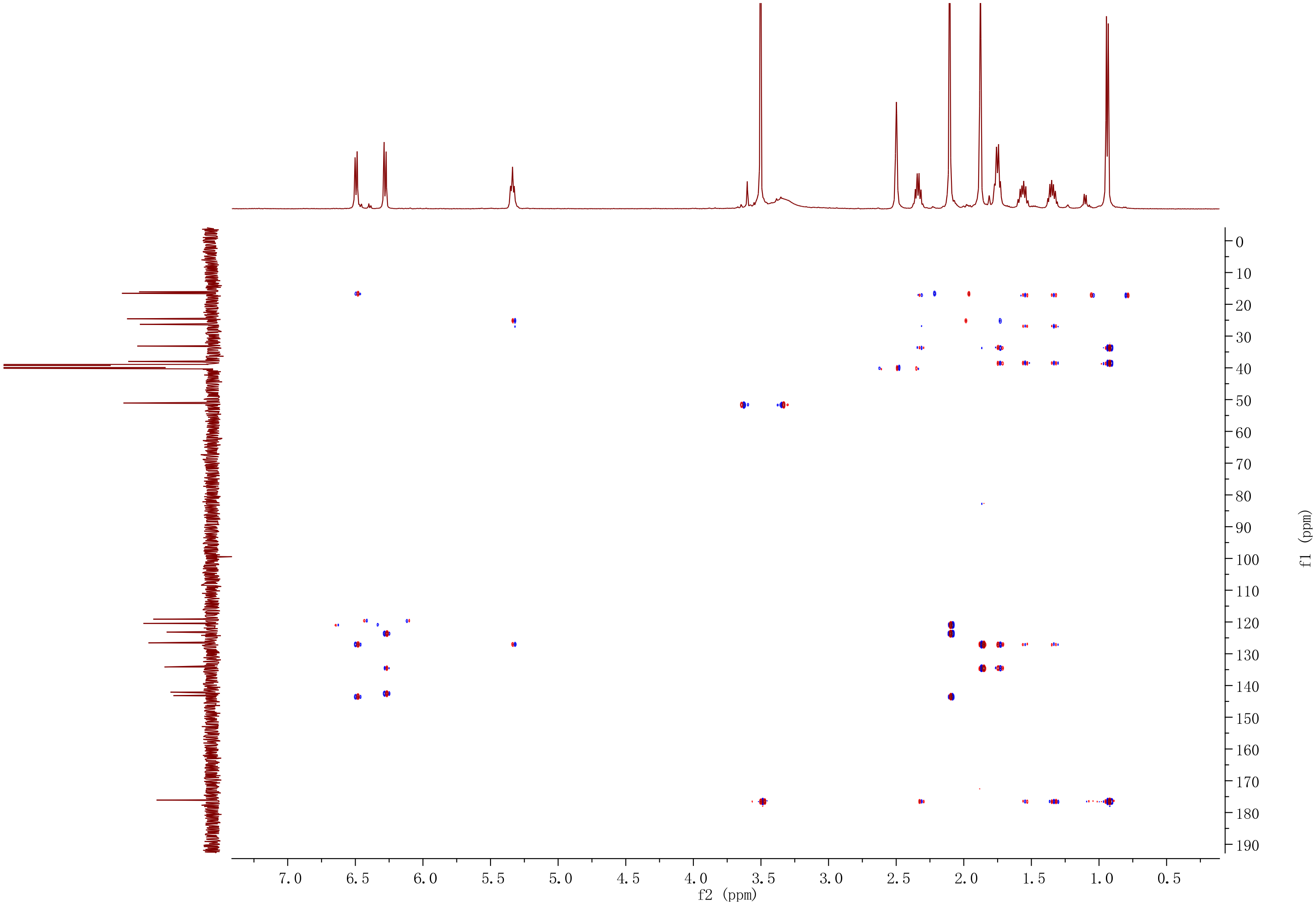

Figure S13.NOESY (DMSO-d_6) spectrum of compound **2**.

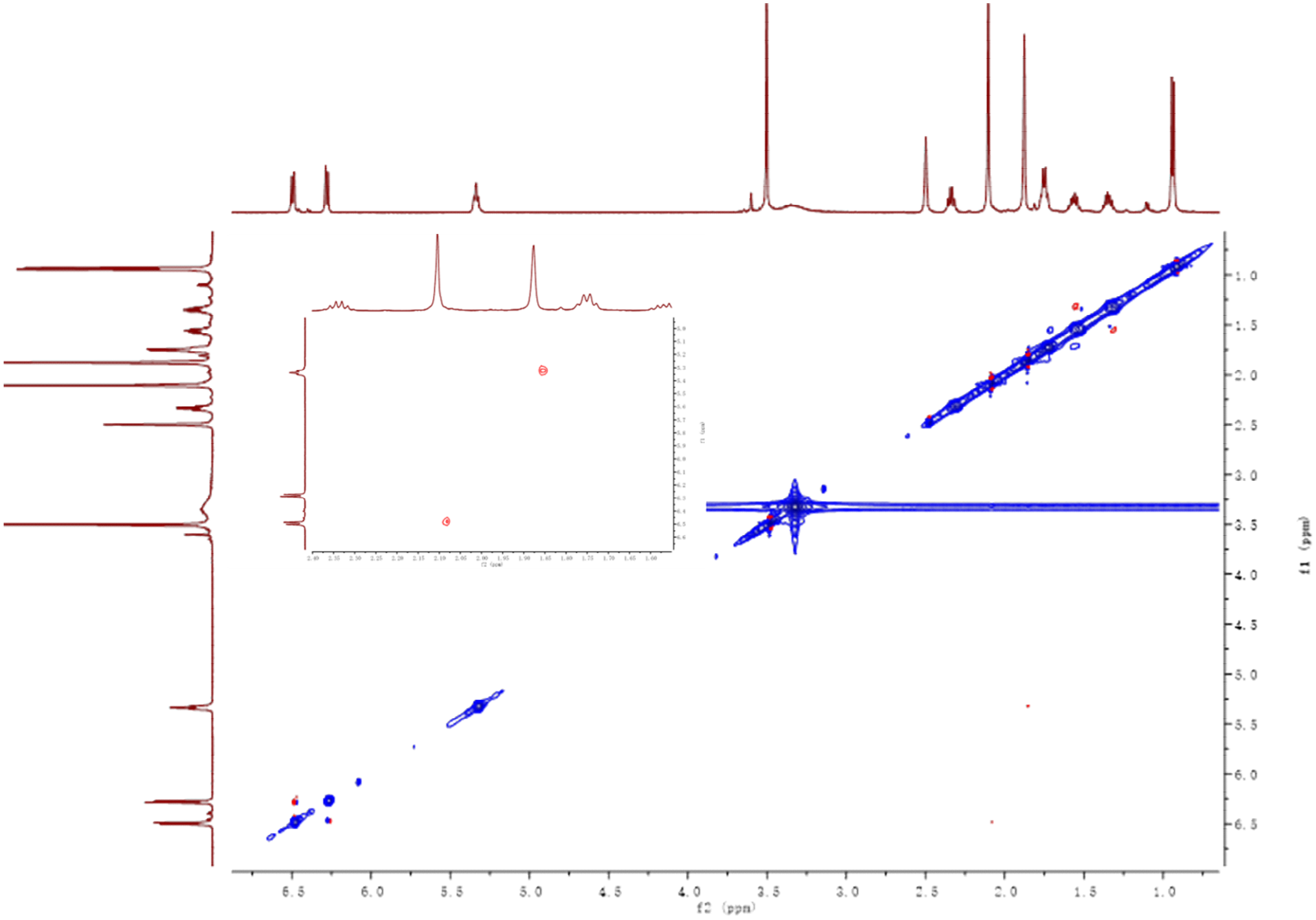

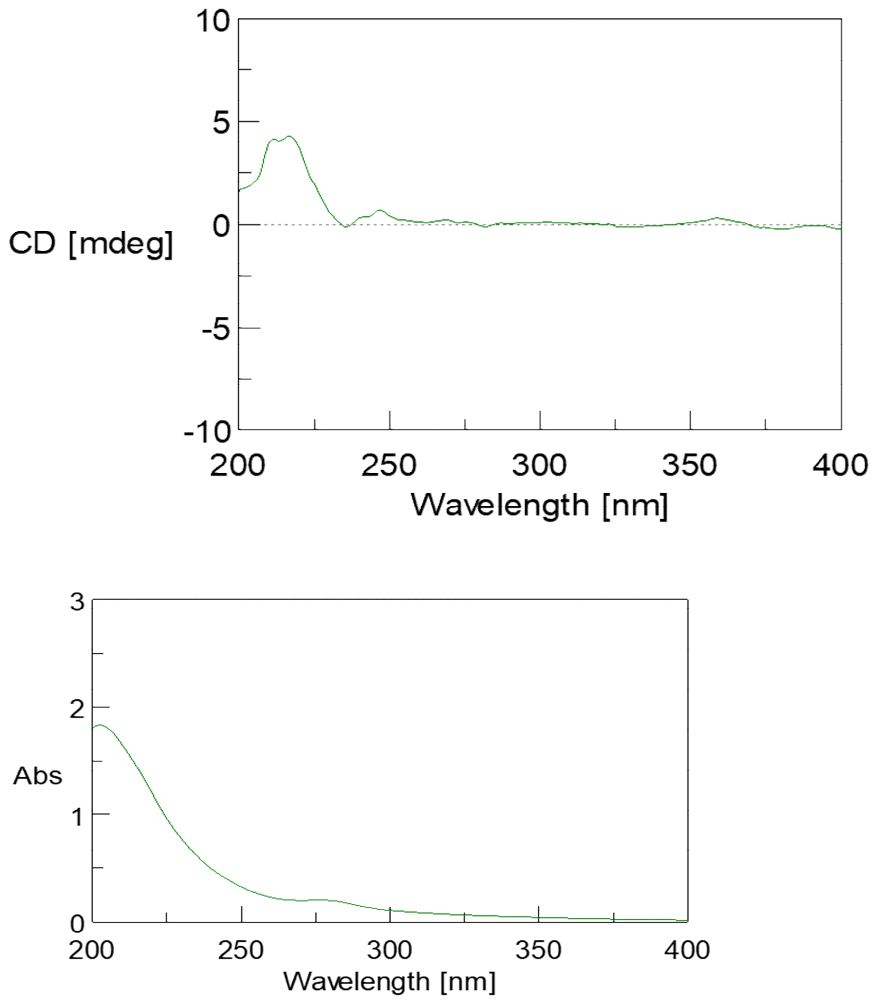

Figure S14. ECD and UV spectra of compound **2**.

Figure S15. HRESI mass spectrum of compound **3**.

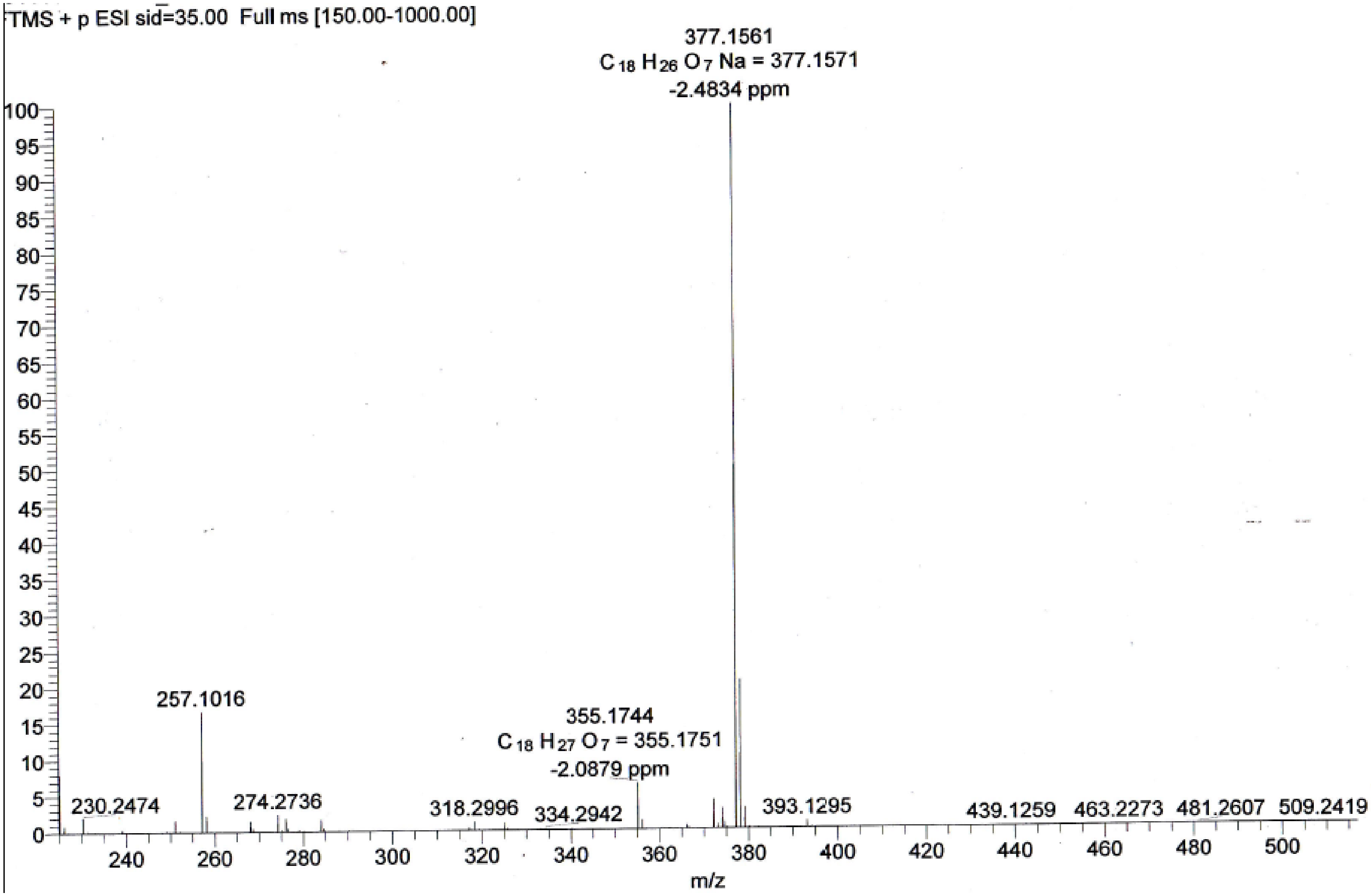

Figure S16. ^{1}H NMR (500 MHz, DMSO-d_6) spectrum of compound **3**.

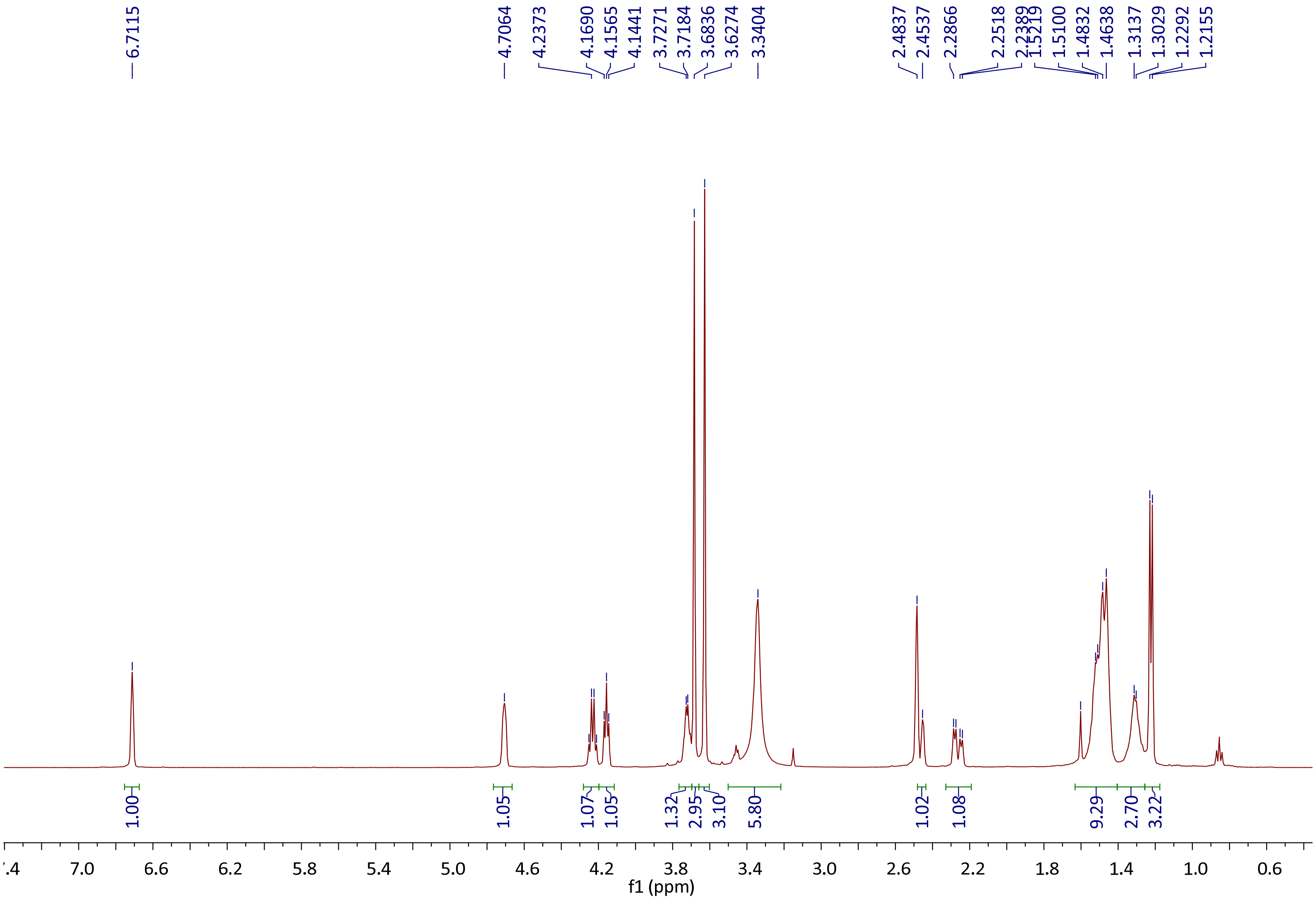

Figure S17. ^{13}C NMR (125 MHz, DMSO-d_6) and DEPT spectra of compound **3**.

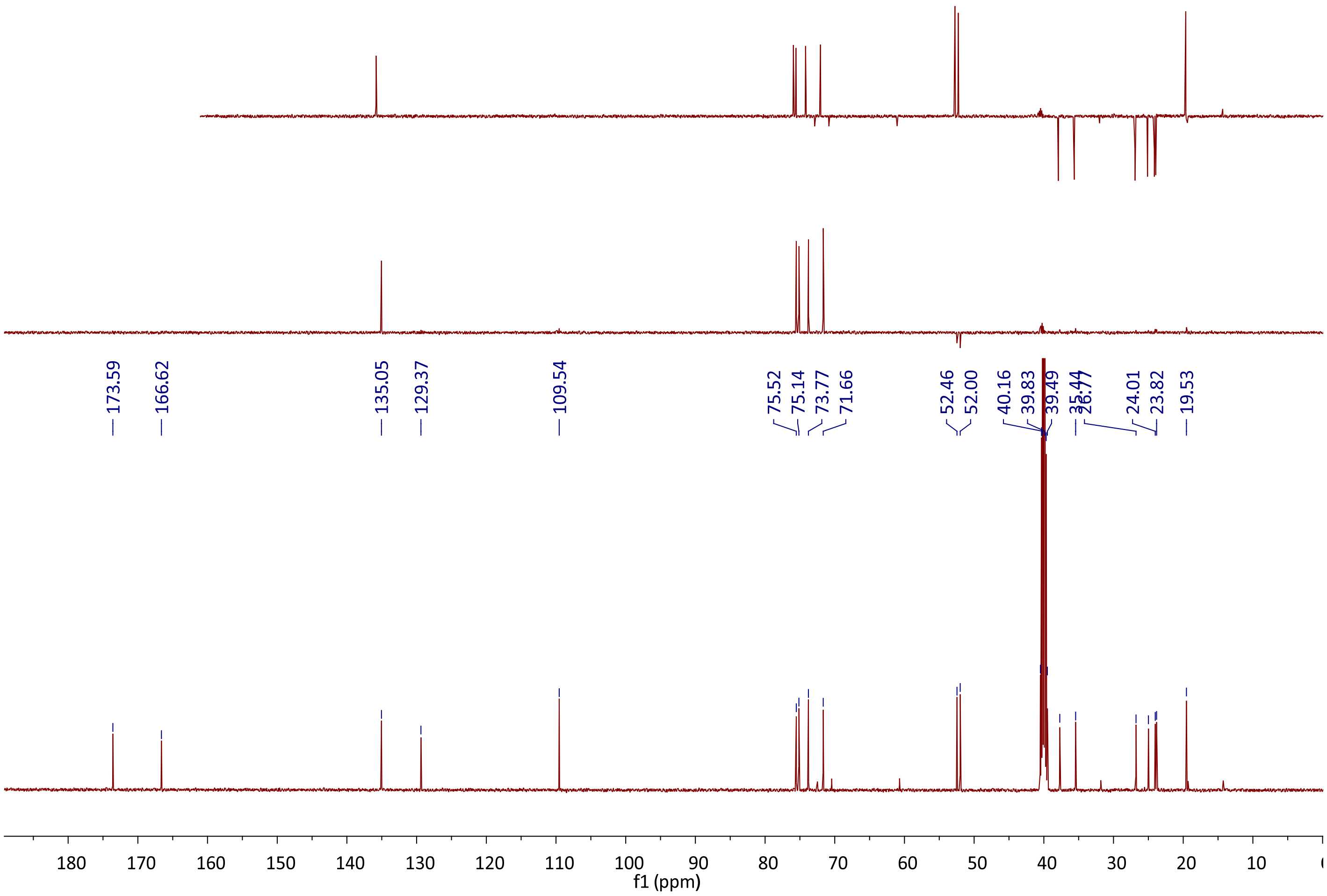

Figure S18. COSY (DMSO-d_6) spectrum of compound **3**.

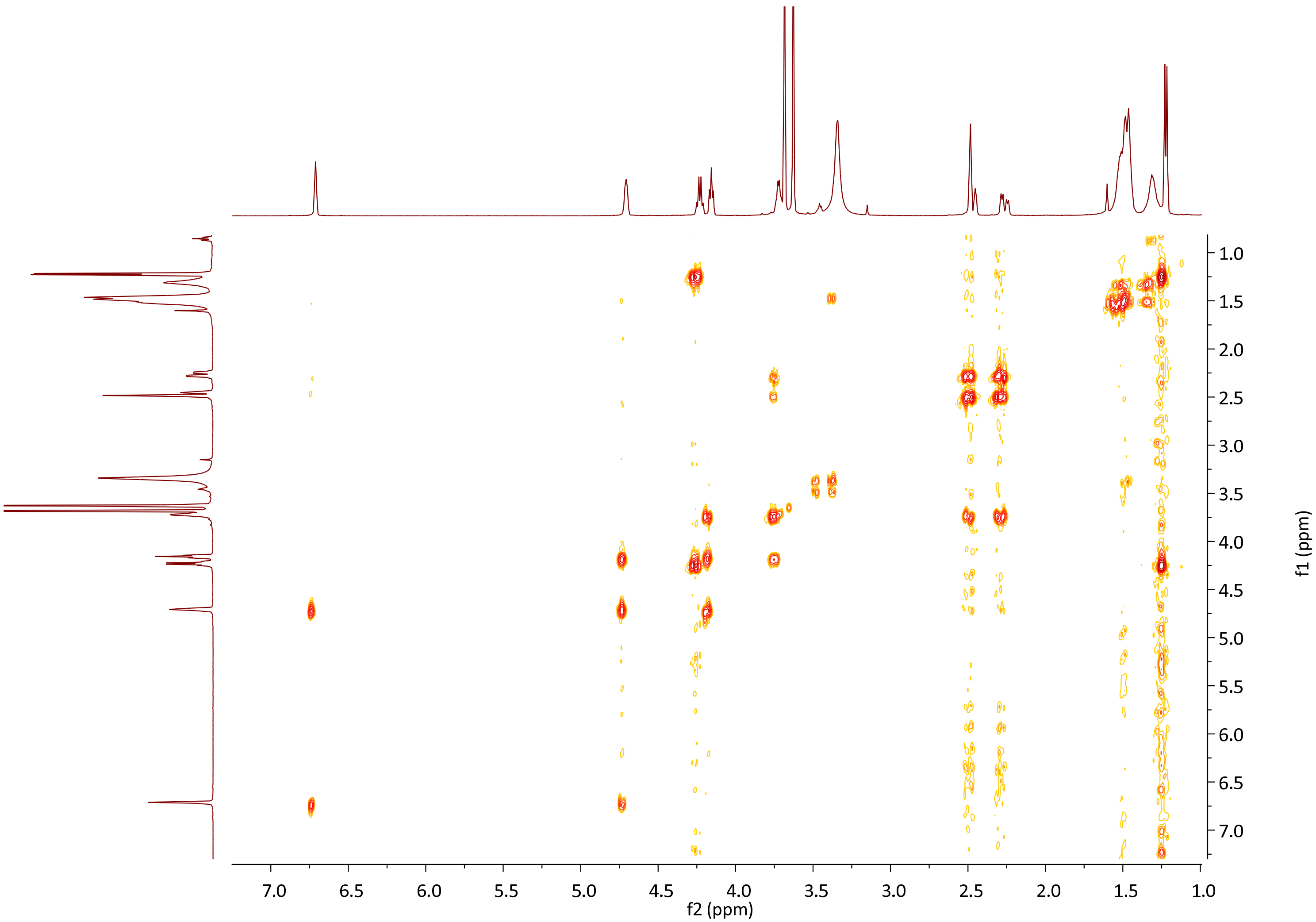

Figure S19. HSQC (DMSO-d_6) spectrum of compound **3**.

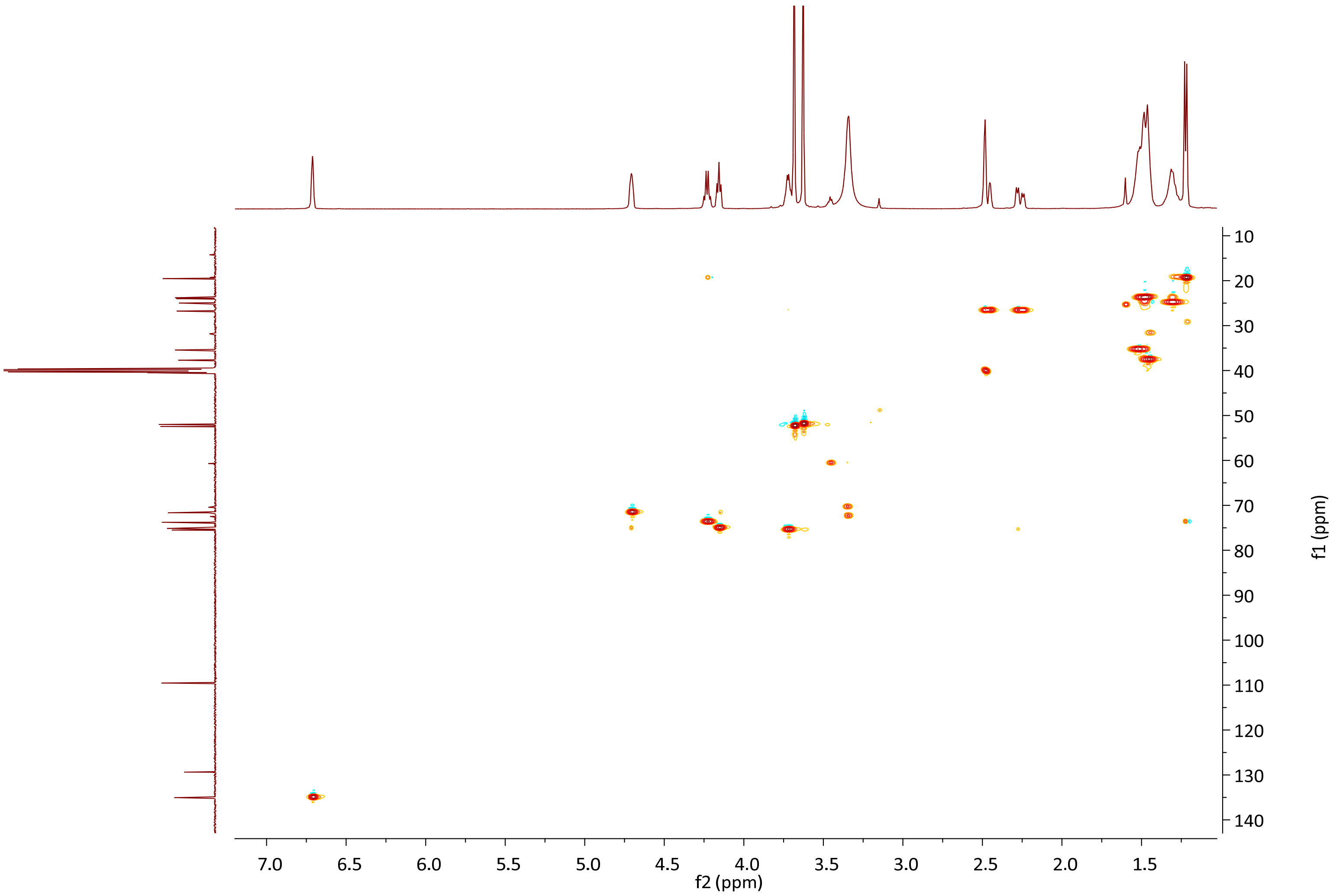

Figure S20. HMBC (DMSO-d_6) spectrum of compound **3**.

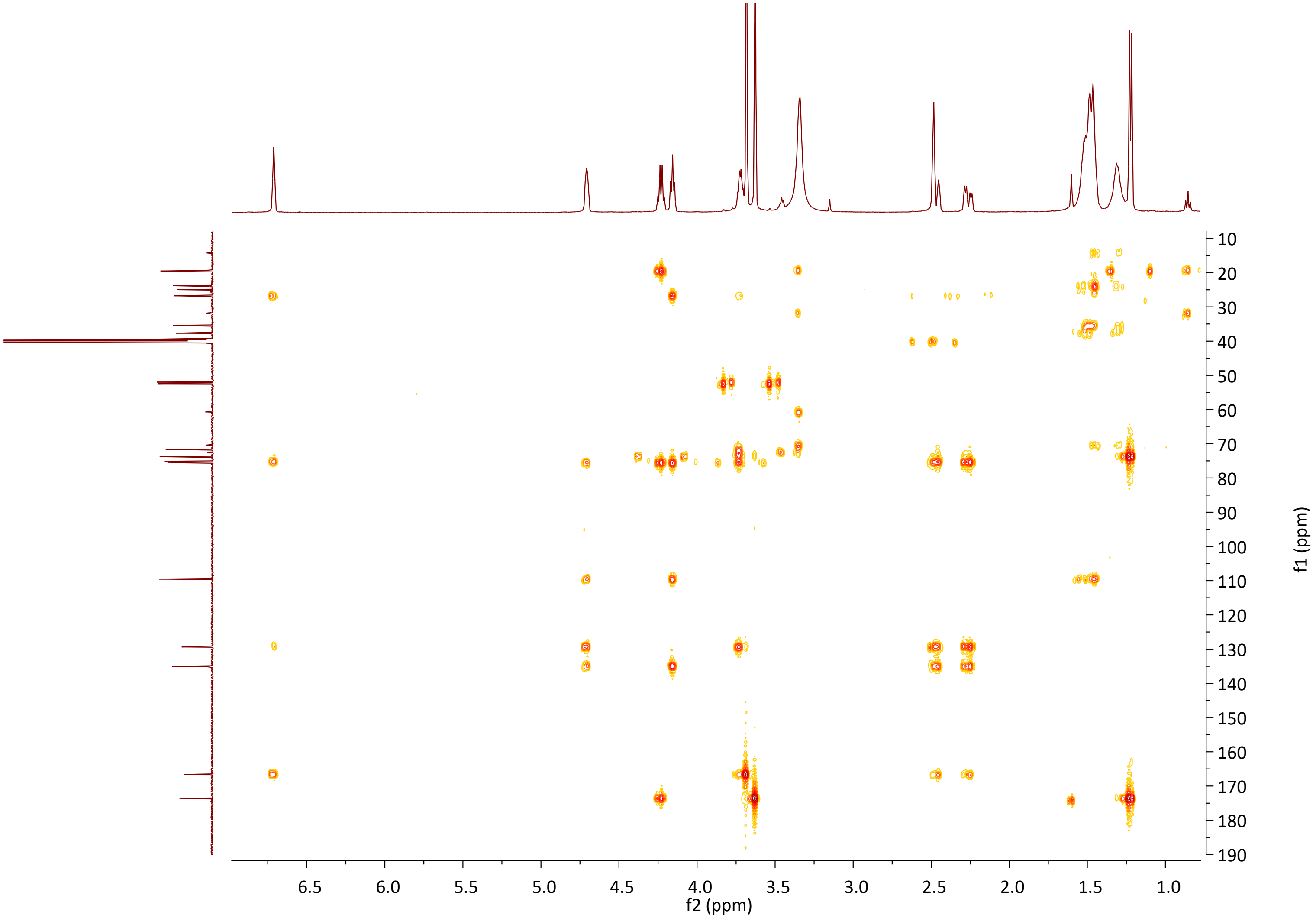

Figure S21. NOESY (DMSO-d_6) spectrum of compound **3**.

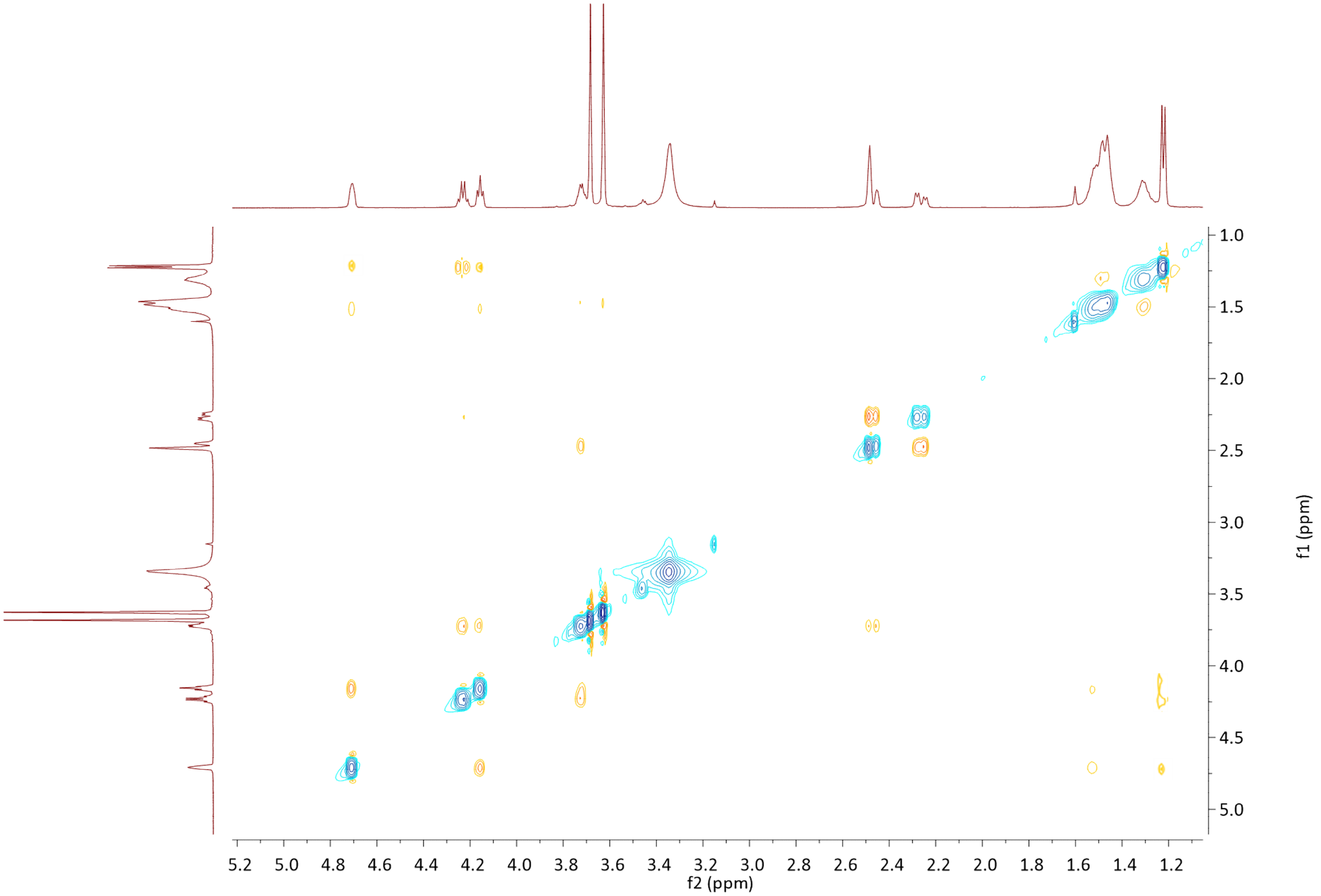

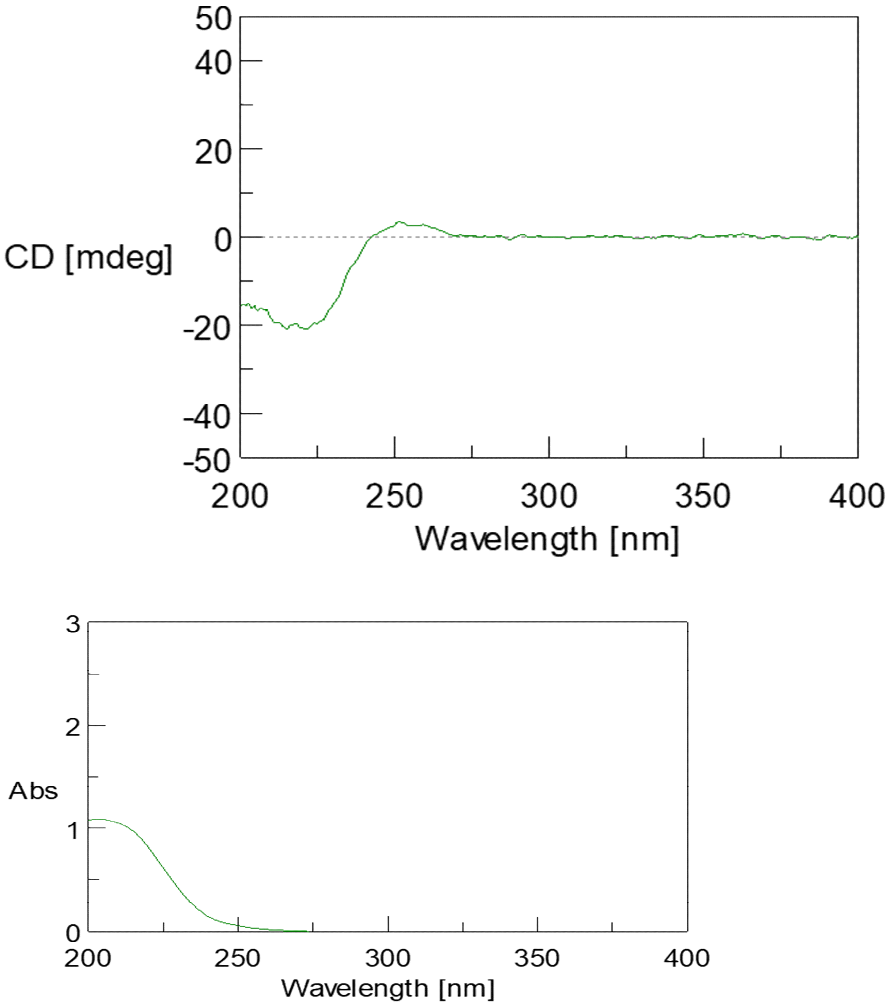

Figure S22. ECD and UV spectra of compound **3**.

Figure S23. ^{1}H NMR (500 MHz, CD_3OD) spectrum of compound **4**.

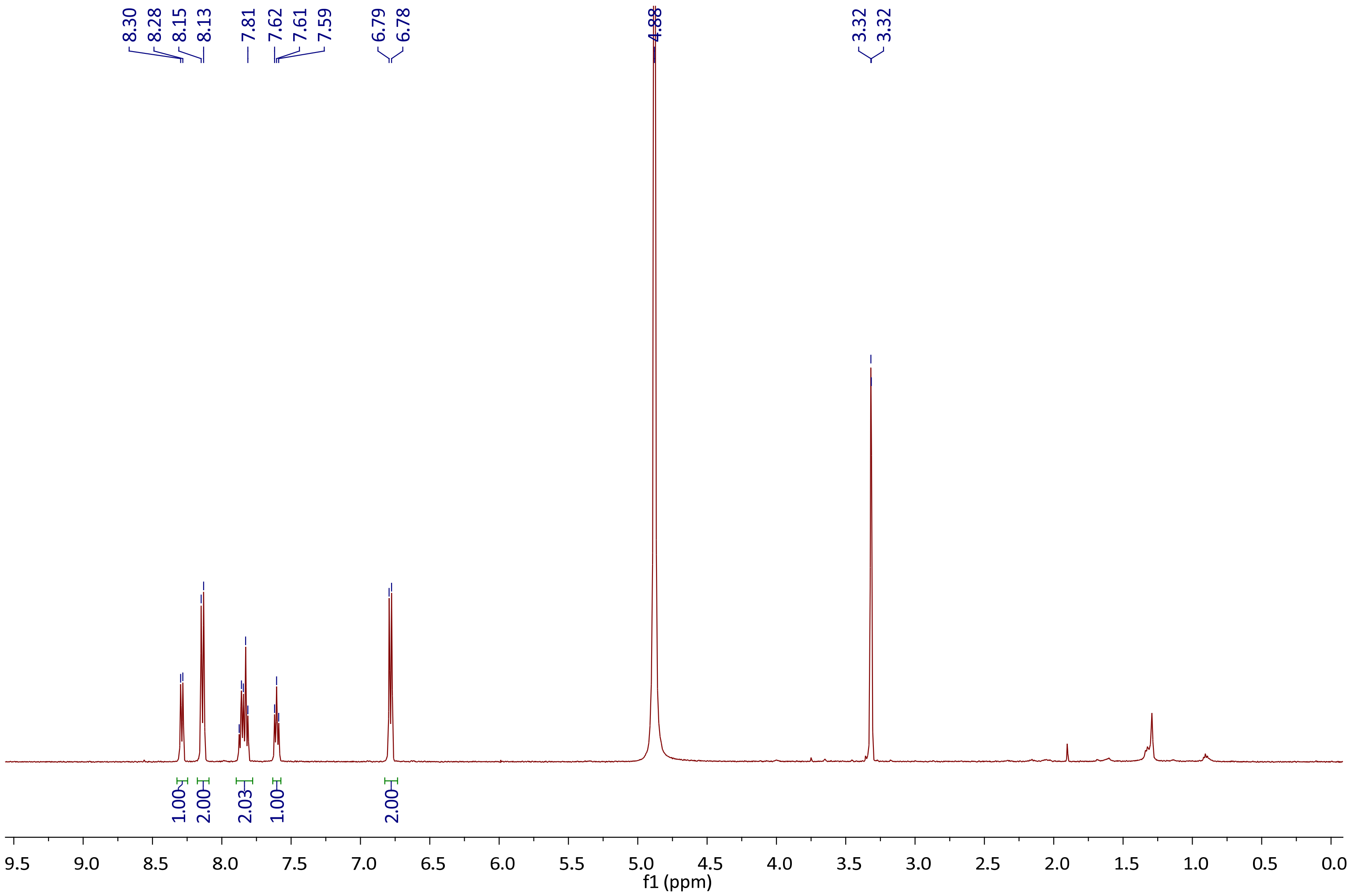

Figure S24. ^{13}C NMR (125 MHz, CD_3OD) spectrum of compound **4**.

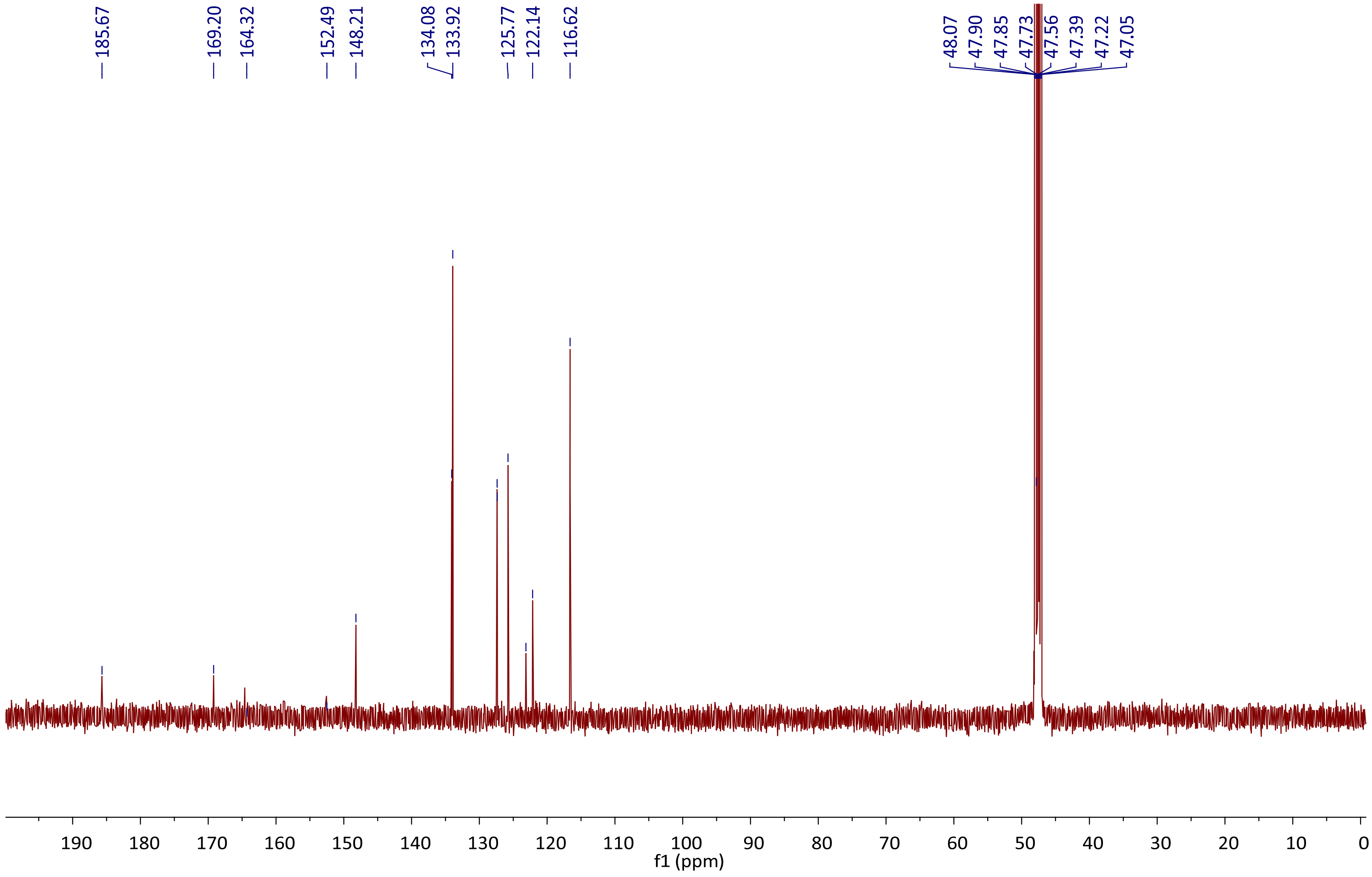

Figure S25. ^{1}H NMR (500 MHz, DMSO-d_6) spectrum of compound **5**.

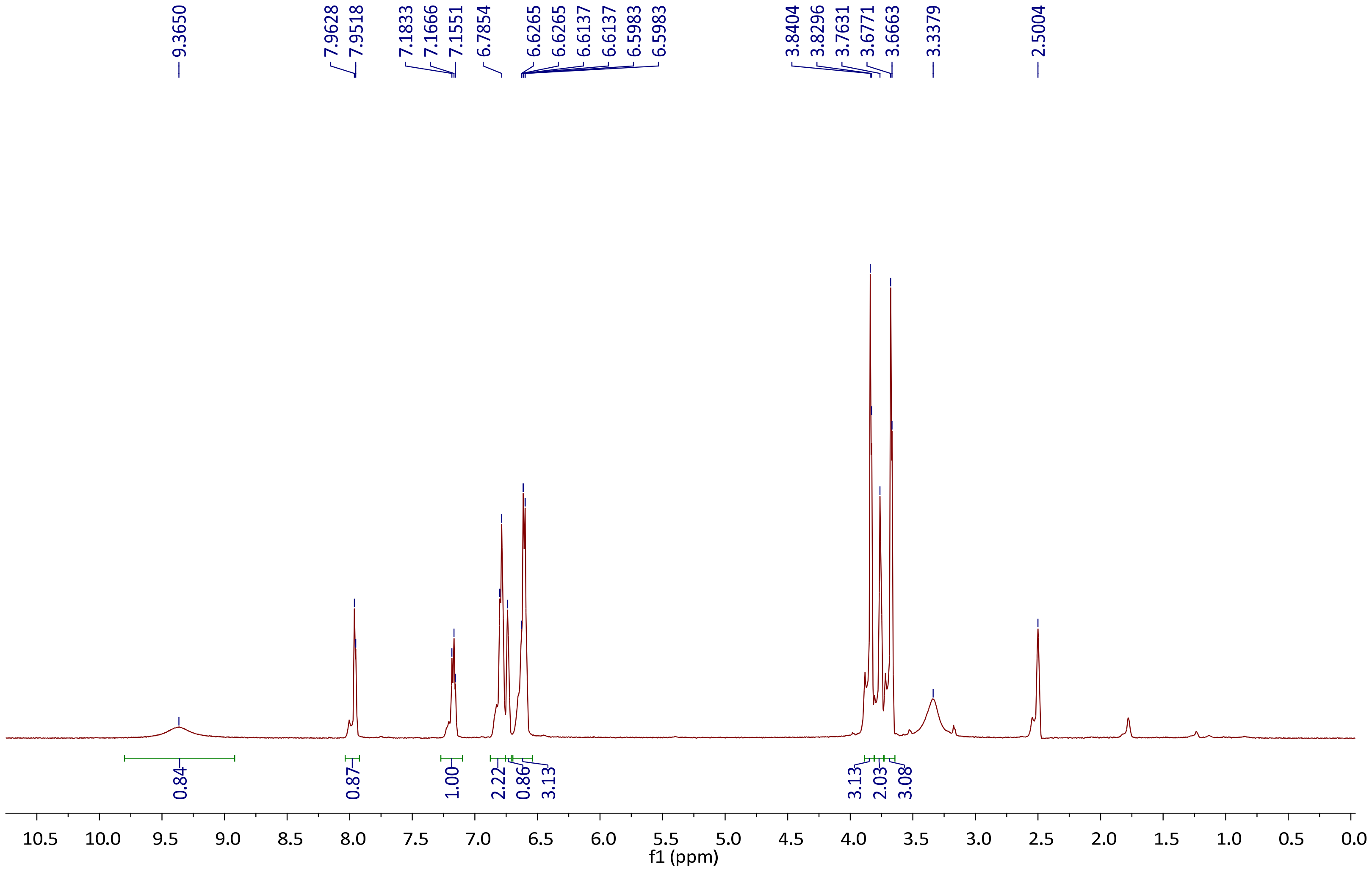

Figure S26. ^{13}C NMR (125 MHz, DMSO-d_6) spectrum of compound **5**.

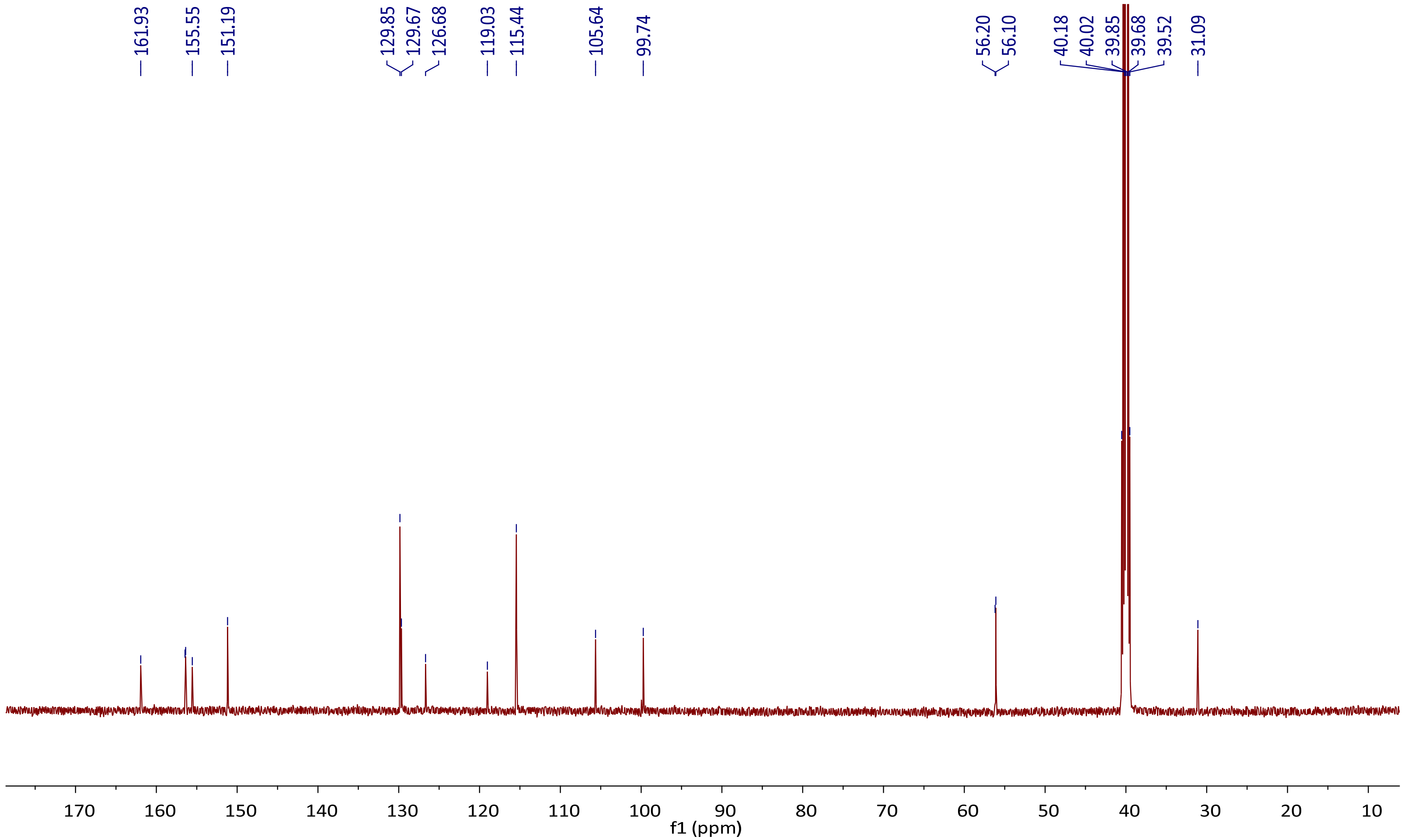

Supplement: Supplementary file 1 [file molecules-24-04596-s001.pdf]
